# Antidepressant use and risk of cardiovascular outcomes in people aged 20 to 64: cohort study using primary care database

**DOI:** 10.1136/bmj.i1350

**Published:** 2016-03-22

**Authors:** Carol Coupland, Trevor Hill, Richard Morriss, Michael Moore, Antony Arthur, Julia Hippisley-Cox

**Affiliations:** 1 Division of Primary Care, School of Medicine, University of Nottingham, Nottingham NG7 2RD, UK; 2 Institute of Mental Health, Nottingham NG8 1BB, UK; 3 University of Southampton Medical School, Primary Care and Population Sciences, Aldermoor Health Centre, Southampton SO16 5ST, UK; 4 School of Health Sciences, Faculty of Medicine and Health Sciences, University of East Anglia, Norwich NR4 7TJ, UK

## Abstract

**Objective** To assess associations between different antidepressant treatments and rates of three cardiovascular outcomes (myocardial infarction, stroke or transient ischaemic attack, and arrhythmia) in people with depression.

**Design** Cohort study.

**Setting** UK general practices contributing to the QResearch primary care database.

**Participants** 238 963 patients aged 20 to 64 years with a first diagnosis of depression between 1 January 2000 and 31 July 2011.

**Exposures** Antidepressant class (tricyclic and related antidepressants, selective serotonin reuptake inhibitors, other antidepressants), dose, duration of use, and commonly prescribed individual antidepressant drugs.

**Main outcome measures** First diagnoses of myocardial infarction, stroke or transient ischaemic attack, and arrhythmia during five years’ follow-up. Cox proportional hazards models were used to estimate hazard ratios, adjusting for potential confounding variables.

**Results** During five years of follow-up, 772 patients had a myocardial infarction, 1106 had a stroke or transient ischaemic attack, and 1452 were diagnosed as having arrhythmia. No significant associations were found between antidepressant class and myocardial infarction over five years’ follow-up. In the first year of follow-up, patients treated with selective serotonin reuptake inhibitors had a significantly reduced risk of myocardial infarction (adjusted hazard ratio 0.58, 95% confidence interval 0.42 to 0.79) compared with no use of antidepressants; among individual drugs, fluoxetine was associated with a significantly reduced risk (0.44, 0.27 to 0.72) and lofepramine with a significantly increased risk (3.07, 1.50 to 6.26). No significant associations were found between antidepressant class or individual drugs and risk of stroke or transient ischaemic attack. Antidepressant class was not significantly associated with arrhythmia over five years’ follow-up, although the risk was significantly increased during the first 28 days of treatment with tricyclic and related antidepressants (adjusted hazard ratio 1.99, 1.27 to 3.13). Fluoxetine was associated with a significantly reduced risk of arrhythmia (0.74, 0.59 to 0.92) over five years, but citalopram was not significantly associated with risk of arrhythmia even at high doses (1.11, 0.72 to 1.71 for doses ≥40 mg/day).

**Conclusions** This study found no evidence that selective serotonin reuptake inhibitors are associated with an increased risk of arrhythmia or stroke/transient ischaemic attack in people diagnosed as having depression between the ages of 20 to 64 or that citalopram is associated with a significantly increased risk of arrhythmia. It found some indication of a reduced risk of myocardial infarction with selective serotonin reuptake inhibitors, particularly fluoxetine, and of an increased risk with lofepramine.

## Introduction

Depression is a common and debilitating condition, which is often treated with antidepressants. Depression increases the risk of cardiovascular outcomes, but controversy exists as to whether use of antidepressants, particularly selective serotonin reuptake inhibitors, increases or reduces the risk.^1^ ^2^ This is important because antidepressants are one of the most commonly prescribed types of drug worldwide, and their use is increasing.^3^ ^4^ ^5^ In the United States, antidepressants were the third most commonly used prescription drug in 2005-08, and their use had increased by almost 400% compared with 1988-94^6^; in England, more than 53 million prescriptions for antidepressants were issued in 2013,^7^ nearly a twofold increase compared with a decade earlier.^8^ More than half (54%) of the prescriptions in England in 2013 were for selective serotonin reuptake inhibitors, including nearly 14 million prescriptions for the most commonly prescribed antidepressant citalopram.

Theoretically, antidepressants such as selective serotonin reuptake inhibitors may have effects on coagulation, and some studies have explored their cardioprotective effect. These studies have tended to be underpowered and explored outcomes in secondary care or other selected populations. Randomised controlled trials of antidepressants tend to be short term and underpowered to detect effects on cardiovascular outcomes, and observational studies of cardiovascular outcomes show conflicting results and many have not accounted for depression and so are susceptible to indication biases. The observational studies have either been restricted to or predominantly included older people, so uncertainty exists about associations in a younger age group, although antidepressants are often prescribed for depression in adults of working age. Antidepressants may have differential effects on cardiovascular outcomes according to age. A meta-analysis of 13 observational studies found that use of selective serotonin reuptake inhibitors was associated with a 40% increased risk of stroke, but this was significant only in studies restricted to older age groups and no significantly increased risk was seen in studies with no age restriction, although none of the studies specifically focused on a younger age group.^9^ Similarly, for myocardial infarction, uncertainty exists about an association with selective serotonin reuptake inhibitors. A large observational study in people aged 65 and over with depression found an increased risk of myocardial infarction with selective serotonin reuptake inhibitors,^10^ whereas other studies in broader age groups have found no association or reduced risks,^11^ ^12^ ^13^ which could be a result of differing age ranges or indication biases.

The US Food and Drug Administration (FDA) issued a drug safety communication in 2011, stating that citalopram should not be prescribed at doses greater than 40 mg per day, based on findings of QT interval prolongation in a study of 119 participants who received different doses of citalopram.^14^ The European Medicines Agency issued a similar safety warning in 2011. Further studies have reported QT interval prolongation with citalopram and also with some other antidepressants such as escitalopram and amitriptyline.^15^ ^16^ QT interval prolongation can lead to arrhythmias including potentially fatal torsades de pointes,^17^ but few studies have specifically assessed risk of arrhythmia for different antidepressant drugs. A cohort study in predominantly older men of two different selective serotonin reuptake inhibitor antidepressants found significantly lower risks of arrhythmia for doses of citalopram over 40 mg/day compared with doses of 1-20 mg/day, with similar findings for sertraline.^18^ A cohort study based on claims data in the United States found no significant differences in risk of ventricular arrhythmia/sudden death for 20 types of antidepressant drug compared with paroxetine, except for a higher risk in mirtazapine users.^19^

Few observational studies of cardiovascular effects have examined associations with individual drugs, so evidence for specific commonly prescribed antidepressants is lacking, especially in younger people, as is evidence in relation to duration and dose. We therefore carried out a cohort study in people aged 20 to 64 to investigate the associations between different antidepressant drugs and the risk of myocardial infarction, arrhythmia, and stroke/transient ischaemic attack and also examined both dose and duration of use.

## Methods

The cohort study was designed to estimate associations between antidepressant treatment and several different adverse outcomes including arrhythmia, myocardial infarction, and stroke or transient ischaemic attack. Full details of the study design, outcomes, and methods can be found in the study protocol.^20^ Results relating to the epilepsy, suicide, and self harm outcomes have been published previously.^21^ ^22^

### Study cohort

The study cohort was selected from a large primary care database (QResearch, version 34). At the time of the study, the QResearch database contained the anonymised longitudinal health records of more than 12 million patients from more than 600 general practices across the United Kingdom, which record data using the Egton Medical Information Systems (EMIS) medical records computer system. Recorded information includes patients’ characteristics, clinical diagnoses, symptoms, and prescribed drugs.

The cohort included patients with a first computer recorded diagnosis of depression between the ages of 20 and 64 years at the time of diagnosis, from 1 January 2000 to 31 July 2011, as described previously.^22^ We identified patients with a diagnosis of depression by using diagnostic Read codes used in previous studies.^10^ ^23^ ^24^ Read codes are the clinical codes used in general practice in the United Kingdom. Patients were eligible for inclusion if their diagnosis of depression occurred at least 12 months after their registration with a study practice and the installation date of their practice’s EMIS computer system. We restricted our cohort to patients with a first recorded diagnosis of depression so that antidepressant prescribing during follow-up would not be influenced by any previous experiences and preferences that would be difficult to account for in the analyses. We used the 12 month inclusion criterion to ensure that the diagnosis of depression was not a retrospective recording of a previous diagnosis.

We excluded patients with a previous recorded diagnosis of depression; those with a diagnosis of schizophrenia, bipolar disorder, or another type of psychosis; and those who had received prescriptions for lithium or antimanic drugs. We also excluded patients if they had received prescriptions for an antidepressant before the study start date (1 January 2000), before their registration date, before they were aged 20, or more than 36 months before their first recorded diagnosis of depression. Temporary residents were also excluded.

The patient’s study entry date was the earliest of the date of the first recorded diagnosis of depression or the date of the first prescription for an antidepressant. Participants in the cohort were followed up until the earliest of date of death, date of leaving the practice, or the end of the follow-up period (1 August 2012).

### Outcomes

The three outcomes for these analyses were arrhythmia, myocardial infarction, and stroke or transient ischaemic attack. We identified patients with these outcomes if they were recorded either on their general practice record using the relevant Read codes or on their linked Office of National Statistics cause of death record using ICD (international classification of diseases) diagnostic codes, based on codes used in previous studies,^25^ ^26^ ^27^ as listed in the web appendix. For the analysis of each separate outcome, we considered only the first event and excluded patients with a previous diagnosis of the outcome recorded at baseline.

### Exposures

We extracted information on all prescriptions for antidepressants during follow-up. We calculated the duration of each prescription by dividing the number of tablets prescribed by the number to be taken each day.^22^

For the main analyses, we grouped antidepressant drugs according to the four main classes in the *British National Formulary*: tricyclic and related antidepressants, selective serotonin reuptake inhibitors, monoamine oxidase inhibitors, and other antidepressants. We classified prescriptions for different antidepressant drugs on the same date as combined prescriptions.

We calculated the daily dose of each prescription by multiplying the number of tablets to be taken each day by the dose of each tablet, and we converted this to a defined daily dose to enable comparison of doses between antidepressant classes, using values assigned by the World Health Organization’s Collaborating Centre for Drug Statistics Methodology (www.whocc.no/atc_ddd_index). For some prescriptions, the dosing instructions were missing or not sufficiently detailed to allow calculation of a daily dose (<5% of total prescriptions). We also assessed the 11 most frequently prescribed individual antidepressant drugs.^10^ ^19^ ^22^

### Confounding variables

We extracted data on variables considered to be potential risk factors for the cardiovascular outcomes or associated with the likelihood of receiving a particular antidepressant treatment, based on our previous study of antidepressants in people aged 65 or over.^10^ These were age at study entry (continuous); sex; year of diagnosis of depression (continuous); severity of index diagnosis of depression (categorised as mild, moderate, or severe, using the classification of Read codes for depression published by Martinez and colleagues^23^ and additional classification by a member of the study team (RM) of some Read codes for depression used in our study^21^ but not included in the study by Martinez); deprivation (Townsend deprivation score corresponding to the patient’s postcode, in fifths); smoking status (non-smoker, ex-smoker, light smoker (1-9 cigarettes/day), moderate smoker (10-19 cigarettes/day), heavy smoker (≥20 cigarettes/day), not recorded); alcohol intake (none, trivial (<1 unit/day), light (1-2 units/day), medium (3-6 units/day), heavy (7-9 units/day), very heavy (>9 units/day), not recorded); ethnic group (categorised into a binary variable of white/not recorded or non-white (comprising Indian, Pakistani, Bangladeshi, other Asian, black African, black Caribbean, Chinese, other including mixed)); comorbidities at baseline (individual binary variables for each of coronary heart disease, diabetes, hypertension, cancer, epilepsy/seizures, hypothyroidism, osteoarthritis, rheumatoid arthritis, asthma/chronic obstructive pulmonary disease, osteoporosis, liver disease, renal disease, obsessive-compulsive disorder); and use of other drugs at baseline (individual binary variables for each of antihypertensives, aspirin, statins, anticoagulants, non-steroidal anti-inflammatory drugs, anticonvulsants, hypnotics/anxiolytics, antipsychotics, bisphosphonates, oral contraceptives, hormone replacement therapy). In addition, for the arrhythmia and myocardial infarction outcomes, we adjusted for a diagnosis of stroke or transient ischaemic attack at baseline. We included year of diagnosis of depression as a confounding variable to account for changes in prescribing patterns over time.

### Statistical analysis

We used Cox’s proportional hazards models to estimate associations between the three outcomes and exposure to antidepressant drugs, treating antidepressant exposure as a time varying exposure to allow for patients starting and stopping and also changing between treatments during follow-up. We used robust standard errors to allow for clustering of patients within practices. We excluded patients from the analysis of each outcome if they had the outcome recorded at baseline. We classified patients as exposed to an antidepressant if no gaps of more than 90 days existed between the end of one prescription and the start of the next. If gaps of more than 90 days occurred, patients counted as exposed for the first 90 days and then unexposed for the remaining period. When patients stopped an antidepressant, we classified them as exposed for the first 90 days after the estimated date of stopping, so that outcomes occurring during withdrawal periods would be attributed to the antidepressant. The main analyses were based on the first five years of follow-up after study entry, and patients were censored at the earliest of five years after study entry, date of death, date of leaving the practice, or the end of the follow-up period in these analyses. We selected five years of follow-up for our main analyses as this would incorporate periods of long term treatment and also allow for more events to accrue than a shorter follow-up period would, so increasing the power of the study.

The analyses calculated unadjusted and adjusted hazard ratios for each antidepressant class (tricyclic and related antidepressants, selective serotonin reuptake inhibitors, other antidepressants, combined treatment) compared with periods of no antidepressant treatment. The unexposed reference category included periods of unexposed time in patients treated at other periods of time during follow-up, as well as person years from patients who received no antidepressant treatment throughout follow-up, so the hazard ratios compare rates of the outcomes between exposed and unexposed periods of time throughout follow-up. Patients who received monoamine oxidase inhibitors at any time were excluded from these analyses, as the number in this category was small. We excluded patients with missing deprivation scores from the adjusted analyses. Analyses were carried out for time varying exposures of prescribed daily dose (categorised as ≤0.5, >0.5 and ≤1.0, and >1.0 defined daily doses), and we calculated tests for trend within each drug class by using dose as a continuous variable. Periods of exposure time for which daily dose was missing were excluded from the analysis of dose. We did additional analyses for time since starting treatment (categorised as no use or treatment duration of 1-28 days, 29-84 days, or ≥85 days) and time since stopping treatment (1-28 days, 29-84 days, and 85-182 days after the estimated date of stopping treatment) and for the 11 most commonly prescribed individual antidepressants, as in a previous study.^10^ Individual antidepressants were further categorised by dose (≤1 or >1 defined daily doses), and citalopram was also categorised as ≤20 mg/day, 20-39 mg/day, and ≥40 mg/day for an analysis of the arrhythmia outcome, in light of the FDA’s drug safety communication.^28^

We used Wald’s significance tests to identify significant differences between antidepressant classes and between individual antidepressant drugs. We tested for interactions between class of antidepressant and age and sex. We assessed the proportional hazards assumption by using log minus log plots.

As sensitivity analyses, we repeated the analyses including the entire follow-up period and did an analysis excluding patients who received no antidepressant prescriptions during follow-up.^22^ We repeated our main analyses using selective serotonin reuptake inhibitors as the comparison group for drug class, the middle dose category of selective serotonin reuptake inhibitors as the comparison group for drug dose, and citalopram (the most commonly prescribed antidepressant) as the comparison group for individual antidepressants.

We also did an analysis restricted to the first year of follow-up; we did this because we had some evidence of non-proportional hazards over five years of follow-up, and also this time period more closely reflected the average duration of treatment. As a post hoc analysis, we also estimated adjusted hazard ratios separately using interaction terms for the 0-1 years, 1-3 years, and 3-5 years after the start of follow-up to further investigate changes in hazard ratios over time. We did these analyses for drug class and for only the five most frequently prescribed antidepressants owing to the smaller numbers of events in the later time periods. To examine the effect of adjusting for different confounding variables, we did additional analyses entering the variables in blocks. As a post hoc analysis, we used a stratified Cox model, with stratification by general practice to compare with our main models using robust standard errors to account for clustering by practice.

We calculated absolute risks of the three outcomes over one year, accounting for the confounding variables by using the adjusted hazard ratios from the analyses based on one year of follow-up, according to the method described by Altman et al.^29^

We included all eligible patients in the database in our analyses to maximise power. We used a P value of <0.01 (two tailed) to determine statistical significance. We used Stata (v12.1) for all analyses.

### Patient involvement

No patients were involved in setting the research question or the outcome measures, nor were they involved in the design or implementation of the study. No patients were asked to advise on interpretation or writing up of results. Patient representatives from the QResearch Advisory Board have advised on dissemination of studies using QResearch data, including the use of lay summaries describing the research and its results.

## Results

The initial cohort included 327 235 patients with a first diagnosis of depression made between the ages of 20 and 64, between 1 January 2000 and 31 July 2011. We excluded 88 272 (27.0%) patients because they had schizophrenia, bipolar disorder, or other psychoses; had been treated with lithium or antimanic drugs; or had received a prescription for an antidepressant before the study entry date, before age 20, or more than 36 months before their date of diagnosis of depression. This left 238 963 patients from 687 practices in the final study cohort.

The total length of follow-up was 1 307 326 person years. Among patients in the cohort 123 038 (51.5%) had at least five years of follow-up, with a median of 5.2 (interquartile range 2.5-8.2) years overall. The mean age of patients in the study cohort was 39.5 (SD 11.1) years, and 61% were women (table 1). Townsend deprivation score was missing for 8201 (3.4%) patients.

**Table 1 tbl1:** Characteristics of study cohort (n=238 963) at baseline. Values are numbers (percentages) unless stated otherwise

Characteristic	Value
Female sex	146 028 (61.1)
Mean (SD) age, years	39.5 (11.1)
Ethnic group:	
Recorded	136 624 (57.2)
White/not recorded	227 451 (95.2)
Non-white	11 512 (4.8)
Depression severity (index diagnosis):	
Mild	171 208 (71.7)
Moderate	59 140 (24.8)
Severe	8615 (3.6)
Smoking status*:	
Non-smoker	110 849 (47.5)
Ex-smoker	35 132 (15.1)
Current light smoker	24 104 (10.3)
Current moderate smoker	40 546 (17.4)
Current heavy smoker	22 659 (9.7)
Not recorded	5673
Alcohol consumption*:	
Non-drinker	55 253 (27.2)
Trivial (<1 unit/day)	77 579 (38.2)
Light (1-2 units/day)	51 310 (25.3)
Moderate (3-6 units/day)	14 482 (7.1)
Heavy (7-9 units/day)	2174 (1.1)
Very heavy (>9 units/day)	2391 (1.2)
Not recorded	35 774
Townsend deprivation score in fifths*:	
1 (least deprived)	45 021 (19.5)
2	46 207 (20.0)
3	48 293 (20.9)
4	47 063 (20.4)
5 (most deprived)	44 178 (19.1)
Not recorded	8201
Comorbidities at baseline:	
Coronary heart disease	4109 (1.7)
Diabetes	7371 (3.1)
Hypertension	17 217 (7.2)
Stroke/transient ischaemic attack	1741 (0.7)
Arrhythmia	2373 (1.0)
Any cancer	3810 (1.6)
Asthma/chronic obstructive pulmonary disease	31 816 (13.3)
Epilepsy/seizures	3325 (1.4)
Hypothyroidism	5267 (2.2)
Obsessive-compulsive disorder	494 (0.2)
Osteoarthritis	7228 (3.0)
Osteoporosis	867 (0.4)
Liver disease	698 (0.3)
Renal disease	549 (0.2)
Rheumatoid arthritis	1301 (0.5)
Drugs at baseline:	
Anticonvulsants	2672 (1.1)
Antihypertensives	25 344 (10.6)
Antipsychotics	836 (0.4)
Anticoagulants	1073 (0.5)
Aspirin	7159 (3.0)
Bisphosphonates	854 (0.4)
Hypnotics/anxiolytics	11 354 (4.8)
Non-steroidal anti-inflammatory drugs	12 725 (5.3)
Statins	10 823 (4.5)
Oral contraceptives†	27 396 (18.8)
Hormone replacement therapy†	7207 (4.9)

*Percentages are out of total with recorded values.

†Percentage is for females only.

### Antidepressant treatment during follow-up

During follow-up, 209 476 (87.7%) patients received a total of 3 337 336 antidepressant prescriptions. These comprised 2 379 668 (71.3%) prescriptions for selective serotonin reuptake inhibitors, 533 798 (16.0%) for tricyclic and related antidepressants, and 422 079 (12.7%) for the group of other antidepressants. In addition, 156 patients had received a total of 1791 (0.05%) prescriptions for monoamine oxidase inhibitors. There were 83 784 combined prescriptions for two or more different antidepressant drugs prescribed on the same day. The median duration of treatment during follow-up was 221 (interquartile range 79-590) days.

Among a total of 3 252 633 prescriptions (with combined prescriptions counting as single prescriptions), citalopram was the most commonly prescribed antidepressant (1 023 255 (31.5%) prescriptions) followed by fluoxetine (778 285; 23.9%), and then amitriptyline (236 416; 7.3%). Supplementary table A shows numbers of prescriptions for the 11 most commonly prescribed antidepressants, with information on prescribed daily doses. Distributions of baseline characteristics according to the first antidepressant prescribed for these 11 drugs have been presented in a previous paper.^22^

### Associations with arrhythmia

At baseline, 2373 patients had an existing diagnosis of arrhythmia. We excluded these patients from analysis of the arrhythmia outcome, along with the patients who received prescriptions for monoamine oxidase inhibitors, leaving 236 434 patients in the analysis cohort. During the first five years of follow-up, 1452 new diagnoses of arrhythmia were made, giving an incidence rate of 16.2 per 10 000 person years (20.1 per 10 000 in men and 13.8 per 10 000 in women).

We found no significant associations with arrhythmia (at P<0.01) for any of the drug classes over five years compared with periods of no antidepressant treatment, as shown in table 2, although we saw some indication of a reduced hazard ratio for selective serotonin reuptake inhibitors (adjusted hazard ratio 0.84, 95% confidence interval 0.73 to 0.97; P=0.02) compared with no current use of antidepressants. In a direct comparison with selective serotonin reuptake inhibitors (supplementary table B), we found a significantly increased rate for the group of other antidepressants (adjusted hazard ratio 1.44, 1.12 to 1.85).

**Table 2 tbl2:** Unadjusted and adjusted hazard ratios for arrhythmia by antidepressant class, dose, and duration over 5 years’ follow-up

	No of events	Person years	Unadjusted hazard ratio (95% CI)	Adjusted analysis†	
				Hazard ratio (95% CI)	P value
**Antidepressant class**					
No current use	887	568 365	1.00	1.00	-
TCAs	102	41 208	1.59 (1.29 to 1.96)	1.09 (0.88 to 1.35)	0.46
SSRIs	352	224 985	1.02 (0.89 to 1.18)	0.84 (0.73 to 0.97)	0.02
Other antidepressants	68	28 048	1.55 (1.23 to 1.95)	1.21 (0.96 to 1.54)	0.11
Combined antidepressants	10	4233	1.47 (0.75 to 2.89)	1.07 (0.54 to 2.09)	0.85
**Antidepressant class and dose categories**					
No current use	887	568 365	1.00	1.00	-
TCAs:					
≤0.5 DDD	51	23 506	1.37 (1.03 to 1.82)	0.89 (0.67 to 1.19)	0.44
>0.5 DDD/≤1.0 DDD	26	8400	2.03 (1.39 to 2.96)	1.35 (0.91 to 1.99)	0.14
>1.0 DDD	14	5306	1.66 (0.98 to 2.81)	1.32 (0.77 to 2.26)	0.31
Test for trend§	-	-	-	-	0.15
SSRIs:					
≤0.5 DDD	30	15 995	1.19 (0.82 to 1.71)	0.93 (0.64 to 1.35)	0.71
>0.5 DDD/≤1.0 DDD	236	157 668	0.97 (0.82 to 1.14)	0.79 (0.67 to 0.94)	0.007
>1.0 DDD	75	42 566	1.16 (0.91 to 1.49)	0.98 (0.76 to 1.26)	0.88
Test for trend§	-	-	-	-	0.55
Others:					
≤0.5 DDD	9	4026	1.40 (0.74 to 2.64)	0.98 (0.52 to 1.86)	0.95
>0.5 DDD/≤1.0 DDD	31	13 199	1.52 (1.08 to 2.15)	1.16 (0.81 to 1.65)	0.41
>1.0 DDD	20	8411	1.49 (0.97 to 2.29)	1.28 (0.84 to 1.97)	0.25
Test for trend§	-	-	-	-	0.69
**Antidepressant class by time since starting and stopping treatment**					
No current or recent use	804	510 266	1.00	1.00	-
TCAs:					
First 28 days	23	5482	2.56 (1.64 to 4.02)	1.99 (1.27 to 3.13)	0.003
29-84 days after starting	12	5400	1.36 (0.77 to 2.43)	1.04 (0.58 to 1.87)	0.89
≥85 days after starting	44	18 941	1.52 (1.11 to 2.07)	0.91 (0.67 to 1.25)	0.57
1-28 days after stopping	11	3614	2.04 (1.15 to 3.62)	1.57 (0.86 to 2.86)	0.14
29-84 days after stopping	11	7030	1.02 (0.56 to 1.88)	0.85 (0.46 to 1.56)	0.60
85-182 days after stopping	15	10 711	1.00 (0.60 to 1.66)	0.79 (0.46 to 1.35)	0.39
SSRIs:					
First 28 days	44	20 639	1.31 (0.90 to 1.89)	1.23 (0.85 to 1.79)	0.28
29-84 days after starting	44	27 863	0.95 (0.66 to 1.37)	0.91 (0.63 to 1.32)	0.63
≥85 days after starting	198	127 197	1.04 (0.88 to 1.23)	0.78 (0.66 to 0.92)	0.004
1-28 days after stopping	22	15 685	0.88 (0.58 to 1.36)	0.94 (0.61 to 1.44)	0.76
29-84 days after stopping	41	30 405	0.94 (0.70 to 1.26)	0.94 (0.69 to 1.27)	0.69
85-182 days after stopping	66	46 815	0.97 (0.75 to 1.27)	1.01 (0.77 to 1.33)	0.92
Others:					
First 28 days	7	2776	1.56 (0.75 to 3.23)	1.35 (0.65 to 2.80)	0.42
29-84 days after starting	7	3504	1.44 (0.71 to 2.91)	1.07 (0.50 to 2.30)	0.85
≥85 days after starting	41	16 854	1.52 (1.13 to 2.04)	1.14 (0.85 to 1.54)	0.38
1-28 days after stopping	5	1573	2.00 (0.83 to 4.79)	1.86 (0.78 to 4.46)	0.16
29-84 days after stopping	6	3023	1.29 (0.58 to 2.88)	1.19 (0.54 to 2.65)	0.66
85-182 days after stopping	8	4537	1.16 (0.58 to 2.34)	1.09 (0.54 to 2.21)	0.80

DDD=defined daily dose; SSRI=selective serotonin reuptake inhibitor; TCA=tricyclic and related antidepressant.

*Based on numbers in adjusted analysis.

†Adjusted for age, sex, year of diagnosis of depression, severity of depression, deprivation, smoking status, alcohol intake, ethnic group (white/not recorded or non-white), coronary heart disease, diabetes, hypertension, cancer, epilepsy/seizures, hypothyroidism, osteoarthritis, asthma/chronic obstructive pulmonary disease, stroke/transient ischaemic attack, rheumatoid arthritis, osteoporosis, liver disease, renal disease, obsessive-compulsive disorder, statins, non-steroidal anti-inflammatory drugs, aspirin, antihypertensives, anticonvulsants, hypnotics/anxiolytics, oral contraceptives, hormone replacement therapy, antipsychotics, bisphosphonates, anticoagulants.

‡Daily doses could not be evaluated for some prescriptions.

§Test for trend uses continuous values of dose.

We found no significant trends with dose in the three drug classes (table 2). A significant increase in the rate of arrhythmia occurred in the first 28 days after starting treatment with tricyclic and related antidepressants (adjusted hazard ratio 1.99, 1.27 to 3.13; P=0.003), as well as a significant reduction from 84 days after starting selective serotonin reuptake inhibitors (0.78, 0.66 to 0.92; P=0.004).

In the analysis of the 11 most commonly prescribed drugs, we found significant differences between the drugs overall (P=0.004) but no significant difference between the four tricyclic and related antidepressants (P=0.22) or the five selective serotonin reuptake inhibitors (P=0.39), although we saw a significantly decreased risk for fluoxetine (adjusted hazard ratio 0.74, 0.59 to 0.92; P=0.008) and some indication of an increased risk for lofepramine (1.67, 1.01 to 2.76; P=0.05) compared with periods of no antidepressant treatment (fig 1).

**Figure f1:**
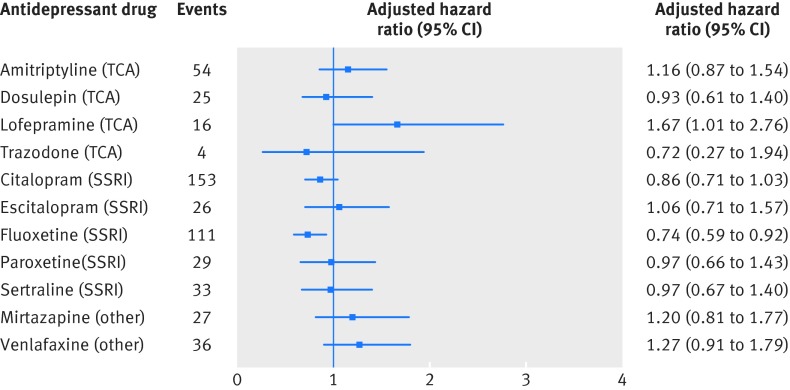
**Fig 1** Adjusted hazard ratios (compared with periods of non-use of antidepressants) for arrhythmia for individual antidepressant drugs over 5 years’ follow-up. SSRI=selective serotonin reuptake inhibitor; TCA=tricyclic and related antidepressant

In an analysis of dose for individual antidepressants (table 3), rates of arrhythmia were not significantly increased for higher doses of citalopram (adjusted hazard ratio 1.08, 0.74 to 1.57, for doses >20 mg/day) or escitalopram (1.06, 0.52 to 2.16, for doses >10 mg/day), but we found a significant increase for lower doses of lofepramine (3.89, 1.92 to 7.90, for doses ≤105 mg/day) and a significantly reduced risk for lower doses of fluoxetine (0.72, 0.56 to 0.91, for doses ≤20 mg/day). Even for doses of citalopram of 40 mg/day or greater, we saw no significantly increased risk (adjusted hazard ratio 1.11, 0.72 to 1.71), although the number of events was small (n=28) (supplementary table C).

**Table 3 tbl3:** Unadjusted and adjusted hazard ratios for arrhythmia by individual drug categorised according to dose for 5 years’ follow-up*

Antidepressant drug	No of events†	Person years†	Unadjusted hazard ratio (95% CI)	Adjusted analysis‡	
				Hazard ratio (95% CI)	P value
No current use	887	568 365	1.00	1.00	-
**Tricyclic and related antidepressants**					
Amitriptyline: ≤1 DDD	41	16 040	-	-	-
Amitriptyline: >1 DDD	4	1442	-	-	-
Dosulepin: ≤1 DDD	23	10 967	-	-	-
Dosulepin: >1 DDD	1	205	-	-	-
Lofepramine: ≤1 DDD	8	961	5.19 (2.55 to 10.54)	3.89 (1.92 to 7.90)	<0.001
Lofepramine: >1 DDD	8	3394	1.49 (0.74 to 2.99)	1.17 (0.58 to 2.39)	0.66
Trazodone: ≤1 DDD	2	2139	-	-	-
Trazodone: >1 DDD	1	19	-	-	-
**Selective serotonin reuptake inhibitors**					
Citalopram: ≤1 DDD	115	72 340	1.04 (0.85 to 1.28)	0.82 (0.66 to 1.01)	0.06
Citalopram: >1 DDD	34	17 854	1.27 (0.88 to 1.83)	1.08 (0.74 to 1.57)	0.70
Escitalopram: ≤1 DDD	18	9068	1.31 (0.81 to 2.12)	1.04 (0.63 to 1.72)	0.88
Escitalopram: >1 DDD	7	3758	1.35 (0.69 to 2.64)	1.06 (0.52 to 2.16)	0.88
Fluoxetine: ≤1 DDD	91	68 345	0.84 (0.66 to 1.07)	0.72 (0.56 to 0.91)	0.007
Fluoxetine: >1 DDD	16	11 072	0.92 (0.56 to 1.53)	0.78 (0.48 to 1.27)	0.32
Paroxetine: ≤1 DDD	19	12 216	0.98 (0.62 to 1.57)	0.84 (0.53 to 1.34)	0.46
Paroxetine: >1 DDD	9	3398	1.72 (0.90 to 3.27)	1.47 (0.77 to 2.84)	0.25
Sertraline: ≤1 DDD	23	11 539	1.31 (0.86 to 2.01)	1.09 (0.70 to 1.68)	0.71
Sertraline: >1 DDD	9	6448	0.89 (0.47 to 1.70)	0.78 (0.41 to 1.49)	0.45
**Others**					
Mirtazapine: ≤1 DDD	20	7533	1.74 (1.13 to 2.70)	1.17 (0.75 to 1.84)	0.49
Mirtazapine: >1 DDD	6	1933	1.94 (0.89 to 4.23)	1.48 (0.67 to 3.26)	0.33
Venlafaxine: ≤1 DDD	18	8432	1.35 (0.86 to 2.12)	1.14 (0.72 to 1.81)	0.57
Venlafaxine: >1 DDD	14	6369	1.38 (0.82 to 2.32)	1.24 (0.74 to 2.08)	0.42

DDD=defined daily dose.

DDD values are amitriptyline 75 mg/day; dosulepin 150 mg/day; lofepramine 105 mg/day; trazodone 300 mg/day; citalopram 20 mg/day; escitalopram 10 mg/day; fluoxetine 20 mg/day; paroxetine 20 mg/day; sertraline 50 mg/day; mirtazapine 30 mg/day; venlafaxine 100 mg/day.

*Results only shown for drugs for which ≥5 events were recorded in both dose categories.

†Based on numbers in adjusted analysis.

‡Adjusted for age, sex, year of diagnosis of depression, severity of depression, deprivation, smoking status, alcohol intake, ethnic group (white/not recorded or non-white), coronary heart disease, diabetes, hypertension, cancer, epilepsy/seizures, hypothyroidism, osteoarthritis, asthma/chronic obstructive pulmonary disease, stroke/transient ischaemic attack, rheumatoid arthritis, osteoporosis, liver disease, renal disease, obsessive-compulsive disorder, statins, non-steroidal anti-inflammatory drugs, aspirin, antihypertensives, anticonvulsants, hypnotics/anxiolytics, oral contraceptives, hormone replacement therapy, antipsychotics, bisphosphonates, anticoagulants.

Adjusted hazard ratios were similar when patients who had not received any prescriptions for antidepressants during follow-up were removed from the analysis (supplementary table D) and when the entire follow-up period was used (supplementary table E), although more associations were significant owing to larger numbers. When we used just the first year of follow-up (table 4), results were similar to the five year analysis, although the hazard ratio for combined antidepressant use was higher (adjusted hazard ratio 3.45, 1.24 to 9.57; P=0.017) and the association with fluoxetine was no longer statistically significant (0.79, 0.55 to 1.13; P=0.19). We found no indication of non-proportional hazards for the arrhythmia outcome; separate results for years 0-1, 1-3, and 3-5 of follow-up are shown in supplementary tables F and G.

**Table 4 tbl4:** Adjusted hazard ratios for arrhythmia, myocardial infarction, and stroke or transient ischaemic attack by antidepressant class, dose, and individual drug over first year of follow-up

	Arrhythmia					Myocardial infarction				Stroke/TIA		
	No of events		Adjusted hazard ratio† (95% CI)	P value		No of events	Adjusted hazard ratio† (95% CI)	P value		No of events	Adjusted hazard ratio† (95% CI)	P value
**Antidepressant class**												
No current use	127		1.00	-		90	1.00	-		113	1.00	-
TCAs	39		1.16 (0.81 to 1.67)	0.42		25	1.09 (0.72 to 1.66)	0.68		33	1.01 (0.69 to 1.49)	0.94
SSRIs	141		0.86 (0.66 to 1.11)	0.24		63	0.58 (0.42 to 0.79)	0.001		118	0.83 (0.63 to 1.09)	0.18
Other antidepressants	20		1.33 (0.84 to 2.12)	0.23		9	0.81 (0.42 to 1.58)	0.54		16	1.15 (0.69 to 1.90)	0.59
Combined antidepressants	5		3.45 (1.24 to 9.57)	0.02		2	1.68 (0.43 to 6.65)	0.46		1	0.69 (0.10 to 4.96)	0.72
**Antidepressant class and dose categories**												
No current use	127	1.00		-		90	1.00	-		113	1.00	-
TCAs:												
≤0.5 DDD	21	0.98 (0.62 to 1.55)		0.92		12	0.86 (0.47 to 1.56)	0.62		18	0.87 (0.54 to 1.41)	0.58
>0.5 DDD/≤1.0 DDD	10	1.76 (0.92 to 3.35)		0.09		4	0.93 (0.35 to 2.50)	0.89		8	1.36 (0.66 to 2.78)	0.41
>1.0 DDD	4	1.22 (0.46 to 3.24)		0.69		3	1.29 (0.41 to 4.04)	0.66		4	1.26 (0.47 to 3.38)	0.65
Test for trend‡	-	-		0.83		-	-	0.47		-	-	0.23
SSRIs:												
≤0.5 DDD	11	0.95 (0.52 to 1.72)		0.85		5	0.76 (0.30 to 1.92)	0.56		7	0.73 (0.34 to 1.56)	0.42
>0.5 DDD/≤1.0 DDD	105	0.81 (0.62 to 1.08)		0.15		43	0.52 (0.37 to 0.73)	<0.001		90	0.81 (0.61 to 1.09)	0.16
>1.0 DDD	21	1.07 (0.65 to 1.76)		0.79		11	0.75 (0.41 to 1.36)	0.34		17	0.99 (0.59 to 1.67)	0.98
Test for trend‡	-	-		0.57		-	-	0.42		-	-	0.47
Others:												
≤0.5 DDD	3	1.06 (0.34 to 3.32)		0.93		2	0.95 (0.23 to 3.96)	0.95		4	1.58 (0.57 to 4.35)	0.38
>0.5 DDD/≤1.0 DDD	13	1.65 (0.91 to 2.98)		0.10		3	0.53 (0.17 to 1.60)	0.26		7	0.95 (0.45 to 1.98)	0.88
>1.0 DDD	2	0.80 (0.20 to 3.20)		0.76		2	1.04 (0.26 to 4.17)	0.95		4	1.76 (0.66 to 4.73)	0.26
Test for trend‡	-	-		0.51		-	-	0.40		-	-	0.72
**Antidepressant drug**												
No current use	130	1.00				90	1.00			113	1.00	
TCAs:												
Amitriptyline	18	1.15 (0.69 to 1.94)		0.59		8	0.75 (0.37 to 1.55)	0.44		15	1.00 (0.59 to 1.70)	1.00
Dosulepin	8	0.73 (0.35 to 1.50)		0.39		8	1.07 (0.53 to 2.18)	0.85		12	1.12 (0.63 to 1.98)	0.70
Lofepramine	8	2.13 (1.05 to 4.33)		0.04		8	3.07 (1.50 to 6.26)	0.002		4	1.15 (0.43 to 3.11)	0.78
Trazodone	3	1.72 (0.53 to 5.56)		0.36		1	0.73 (0.10 to 5.19)	0.76		1	0.56 (0.08 to 3.72)	0.55
SSRIs:												
Citalopram	56	0.79 (0.57 to 1.10)		0.17		27	0.59 (0.39 to 0.91)	0.017		43	0.73 (0.51 to 1.05)	0.09
Escitalopram	9	1.01 (0.47 to 2.16)		0.99		4	0.67 (0.25 to 1.82)	0.43		5	0.63 (0.26 to 1.53)	0.31
Fluoxetine	48	0.79 (0.55 to 1.13)		0.19		18	0.44 (0.27 to 0.72)	0.001		56	1.06 (0.76 to 1.50)	0.72
Paroxetine	13	1.10 (0.61 to 1.99)		0.74		3	0.38 (0.12 to 1.22)	0.10		7	0.63 (0.28 to 1.38)	0.25
Sertraline	15	1.21 (0.71 to 2.07)		0.48		10	1.18 (0.64 to 2.20)	0.59		7	0.63 (0.30 to 1.35)	0.24
Others:												
Mirtazapine	8	1.20 (0.57 to 2.53)		0.62		5	0.91 (0.37 to 2.24)	0.84		12	1.85 (1.01 to 3.37)	0.04
Venlafaxine	11	1.64 (0.88 to 3.08)		0.12		4	0.89 (0.33 to 2.39)	0.81		3	0.51 (0.16 to 1.57)	0.24
All other antidepressants	3	0.90 (0.30 to 2.69)		0.85		1	0.46 (0.06 to 3.35)	0.44		2	0.64 (0.15 to 2.63)	0.53
Combined antidepressants	5	3.44 (1.24 to 9.55)		0.02		2	1.68 (0.43 to 6.64)	0.46		1	0.70 (0.10 to 4.97)	0.72

SSRI=selective serotonin reuptake inhibitor; TCA=tricyclic and related antidepressant; TIA=transient ischaemic attack.

*Based on numbers in adjusted analysis.

†Adjusted for age, sex, year of diagnosis of depression, severity of depression, deprivation, smoking status, alcohol intake, ethnic group (white/not recorded or non-white), coronary heart disease, diabetes, hypertension, cancer, epilepsy/seizures, hypothyroidism, osteoarthritis, asthma/chronic obstructive airways disease, stroke/transient ischaemic attack (except for the stroke/TIA outcome), rheumatoid arthritis, osteoporosis, liver disease, renal disease, obsessive-compulsive disorder, statins, non-steroidal anti-inflammatory drugs, aspirin, antihypertensives, anticonvulsants, hypnotics/anxiolytics, oral contraceptives, hormone replacement therapy, antipsychotics, bisphosphonates, anticoagulants.

‡Test for trend uses continuous values of dose.

### Associations with myocardial infarction

At baseline, 1790 patients had a previous diagnosis of myocardial infarction recorded. We excluded these patients from analysis of the myocardial infarction outcome, along with the patients who received monoamine oxidase inhibitors, leaving 237 017 patients in the analysis cohort. During the first five years of follow-up, 772 new diagnoses of myocardial infarction were made, giving an incidence rate of 8.6 per 10 000 person years (16.2 per 10 000 in men and 3.9 per 10 000 in women).

We found no significant association between antidepressant class and myocardial infarction over five years in the adjusted analysis (table 5) and no significant trends with dose. No clear pattern in risk according to different periods of time after starting or stopping antidepressant drugs was apparent, although rates were increased from 28 days after stopping tricyclic and related antidepressants.

**Table 5 tbl5:** Unadjusted and adjusted hazard ratios for myocardial infarction by antidepressant class, dose, and duration over 5 years’ follow-up

	No of events*	Person years*	Unadjusted hazard ratio (95% CI)	Adjusted analysis†	
				Hazard ratio (95% CI)	P value
**Antidepressant class**					
No current use	469	570 843	1.00	1.00	
TCAs	63	41 295	1.83 (1.44 to 2.33)	1.20 (0.94 to 1.52)	0.14
SSRIs	182	225 863	1.02 (0.86 to 1.22)	0.85 (0.71 to 1.00)	0.06
Other antidepressants	33	28 144	1.39 (0.98 to 1.98)	1.00 (0.70 to 1.42)	0.98
Combined antidepressants	3	4224	0.84 (0.27 to 2.59)	0.57 (0.18 to 1.75)	0.32
**Antidepressant class and dose categories**					
No current use	469	570 843	1.00	1.00	
TCAs:					
≤0.5 DDD	31	23 555	1.59 (1.11 to 2.26)	1.02 (0.72 to 1.45)	0.89
>0.5 DDD/≤1.0 DDD	15	8412	2.15 (1.31 to 3.53)	1.29 (0.78 to 2.13)	0.32
>1.0 DDD	10	5318	2.24 (1.21 to 4.16)	1.59 (0.86 to 2.97)	0.14
Test for trend§	-	-	-	-	0.35
SSRIs:					
≤0.5 DDD	14	16 132	1.12 (0.68 to 1.86)	0.97 (0.57 to 1.63)	0.90
>0.5 DDD/≤1.0 DDD	110	158 252	0.89 (0.72 to 1.11)	0.73 (0.59 to 0.91)	0.005
>1.0 DDD	50	42 683	1.46 (1.11 to 1.92)	1.16 (0.88 to 1.54)	0.30
Test for trend§	-	-	-	-	0.03
Others:					
≤0.5 DDD	9	4041	2.65 (1.38 to 5.10)	1.80 (0.94 to 3.45)	0.08
>0.5 DDD/≤1.0 DDD	8	13 236	0.72 (0.36 to 1.43)	0.51 (0.26 to 1.02)	0.06
>1.0 DDD	11	8440	1.54 (0.86 to 2.78)	1.11 (0.61 to 2.00)	0.74
Test for trend§	-	-	-	-	0.79
**Antidepressant class by time since starting and stopping treatment**					
No current or recent use	416	512 509	1.00	1.00	
TCAs:					
First 28 days	6	5499	1.08 (0.48 to 2.44)	0.83 (0.37 to 1.86)	0.65
29-84 days after starting	5	5414	1.05 (0.44 to 2.51)	0.77 (0.32 to 1.83)	0.55
≥85 days after starting	33	18 957	2.17 (1.56 to 3.00)	1.23 (0.89 to 1.71)	0.21
1-28 days after stopping	5	3627	1.60 (0.66 to 3.86)	1.30 (0.54 to 3.12)	0.56
29-84 days after stopping	13	7056	2.32 (1.32 to 4.06)	1.85 (1.05 to 3.23)	0.03
85-182 days after stopping	20	10 753	2.36 (1.47 to 3.78)	1.89 (1.18 to 3.02)	0.008
SSRIs:					
First 28 days	14	20 710	0.66 (0.35 to 1.25)	0.63 (0.32 to 1.22)	0.17
29-84 days after starting	14	27 967	0.59 (0.34 to 1.02)	0.56 (0.31 to 0.99)	0.05
≥85 days after starting	109	127 711	1.12 (0.91 to 1.38)	0.84 (0.68 to 1.03)	0.10
1-28 days after stopping	20	15 744	1.64 (1.04 to 2.60)	1.66 (1.05 to 2.63)	0.03
29-84 days after stopping	22	30 521	0.96 (0.61 to 1.49)	1.00 (0.64 to 1.58)	0.98
85-182 days after stopping	33	47 004	0.95 (0.65 to 1.38)	0.99 (0.67 to 1.45)	0.95
Others:					
First 28 days	5	2788	1.91 (0.76 to 4.84)	1.52 (0.60 to 3.82)	0.37
29-84 days after starting	2	3514	0.67 (0.17 to 2.66)	0.53 (0.13 to 2.08)	0.36
≥85 days after starting	20	16 908	1.44 (0.90 to 2.29)	0.96 (0.60 to 1.53)	0.87
1-28 days after stopping	1	1580	0.75 (0.11 to 5.35)	0.64 (0.09 to 4.54)	0.65
29-84 days after stopping	4	3036	1.64 (0.62 to 4.37)	1.38 (0.52 to 3.67)	0.52
85-182 days after stopping	5	4557	1.37 (0.56 to 3.33)	1.17 (0.48 to 2.85)	0.72

DDD=defined daily dose; SSRI=selective serotonin reuptake inhibitor; TCA=tricyclic and related antidepressant.

*Based on numbers in adjusted analysis

†Adjusted for age, sex, year of diagnosis of depression, severity of depression, deprivation, smoking status, alcohol intake, ethnic group (white/not recorded or non-white), coronary heart disease, diabetes, hypertension, cancer, epilepsy/seizures, hypothyroidism, osteoarthritis, asthma/chronic obstructive pulmonary disease, stroke/transient ischaemic attack, rheumatoid arthritis, osteoporosis, liver disease, renal disease, obsessive-compulsive disorder, statins, non-steroidal anti-inflammatory drugs, aspirin, antihypertensives, anticonvulsants, hypnotics/anxiolytics, oral contraceptives, hormone replacement therapy, antipsychotics, bisphosphonates, anticoagulants.

‡Daily doses could not be evaluated for some prescriptions.

§Test for trend uses continuous values of dose.

We found no significant associations (at P<0.01) for individual drugs in the adjusted analyses (fig 2) and no significant difference between the five selective serotonin reuptake inhibitors (P=0.27) or the four tricyclic and related antidepressants (P=0.26), although fluoxetine had an adjusted hazard ratio of 0.73 (0.54 to 0.98; P=0.04) and lofepramine had an adjusted hazard ratio of 2.02 (1.14 to 3.59; P=0.02), both compared with periods of no antidepressant treatment.

**Figure f2:**
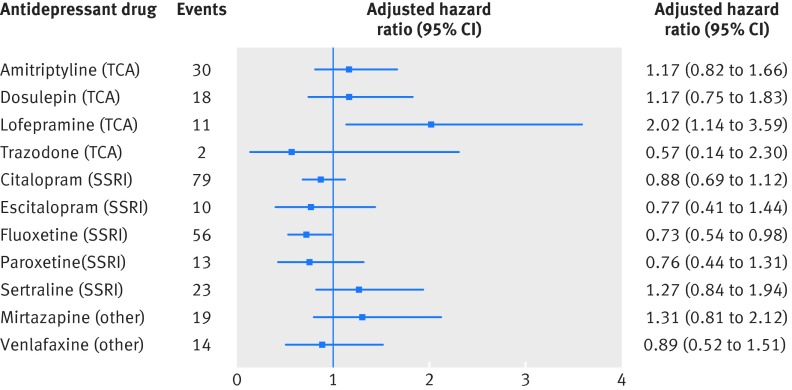
**Fig 2** Adjusted hazard ratios (compared with periods of non-use of antidepressants) for myocardial infarction for individual antidepressant drugs over 5 years’ follow-up. SSRI=selective serotonin reuptake inhibitor; TCA=tricyclic and related antidepressant

Adjusted hazard ratios were similar when patients who had not received any antidepressant prescriptions during follow-up were removed from the analysis (supplementary table H) and when the entire follow-up period was used (supplementary table I). We saw some indication that hazard rates were not proportional over the five years of follow-up, and some differences in the hazard ratios were apparent when the analysis was restricted to the first year of follow-up compared with values over five years. In this one year analysis (table 4), we found a significantly reduced risk for selective serotonin reuptake inhibitors compared with no use of antidepressants (adjusted hazard ratio 0.58, 0.42 to 0.79; P=0.001); although overall no significant difference (at P<0.01) existed between the five selective serotonin reuptake inhibitors (P=0.11) or the four tricyclic and related antidepressants (P=0.03), a significant reduction occurred with fluoxetine (adjusted hazard ratio 0.44, 0.27 to 0.72; P=0.001) and a significant increase with lofepramine (3.07, 1.50 to 6.26; P=0.002). We found no significant associations with selective serotonin reuptake inhibitors in years 1-3 and 3-5 of follow-up (supplementary table F) or with fluoxetine (supplementary table G).

### Associations with stroke/transient ischaemic attack

At baseline, 1741 patients had a diagnosis of stroke or transient ischaemic attack recorded. These patients were excluded from analysis of the stroke/transient ischaemic attack outcome, along with the patients who received monoamine oxidase inhibitors, leaving 237 067 patients in the analysis cohort. During the first five years of follow-up, 1106 new diagnoses of stroke or transient ischaemic attack were made, giving an incidence rate of 12.3 per 10 000 person years (17.3 per 10 000 in men and 9.3 per 10 000 in women).

We found no significant associations between antidepressant class and stroke/transient ischaemic attack over five years and no significant trends (at P<0.01) with dose (table 6). A significant increase in risk occurred during the first 28 days after starting other antidepressants (adjusted hazard ratio 2.72, 1.45 to 5.08; P=0.002) and from 85 to 182 days after stopping tricyclic and related antidepressants (1.82, 1.21 to 2.74; P=0.004). Rates were also increased in the first 84 days after starting tricyclic and related antidepressants, although not significantly (at P<0.01).

**Table 6 tbl6:** Unadjusted and adjusted hazard ratios for stroke or transient ischaemic attack by antidepressant class, dose, and duration over 5 years’ follow-up.

	No of events*		Person years*	Unadjusted hazard ratio (95% CI)	Adjusted analysis†	
					Hazard ratio (95% CI)	P value
**Antidepressant class**						
No current use	610	570 879		1.00	1.00	
TCAs	90	41 109		1.98 (1.56 to 2.52)	1.24 (0.98 to 1.58)	0.08
SSRIs	313	225 600		1.30 (1.12 to 1.51)	1.09 (0.93 to 1.27)	0.28
Other antidepressants	50	28 056		1.71 (1.30 to 2.25)	1.20 (0.91 to 1.60)	0.20
Combined antidepressants	11	4196		2.59 (1.47 to 4.55)	1.54 (0.86 to 2.78)	0.15
**Antidepressant class and dose categories**						
No current use	610	570 879		1.00	1.00	
TCAs:						
≤0.5 DDD	48	23 489		1.85 (1.36 to 2.50)	1.10 (0.81 to 1.49)	0.54
>0.5 DDD/≤1.0 DDD	24	8362		2.62 (1.76 to 3.88)	1.59 (1.06 to 2.37)	0.02
>1.0 DDD	12	5265		2.06 (1.13 to 3.76)	1.52 (0.84 to 2.76)	0.17
Test for trend§	-	-		-	-	0.27
SSRIs:						
≤0.5 DDD	24	16 083		1.37 (0.88 to 2.11)	1.12 (0.72 to 1.73)	0.61
>0.5 DDD/≤1.0 DDD	216	158 042		1.28 (1.09 to 1.52)	1.06 (0.90 to 1.26)	0.47
>1.0 DDD	66	42 676		1.44 (1.12 to 1.87)	1.22 (0.94 to 1.59)	0.14
Test for trend§	-	-		-	-	0.57
Others:						
≤0.5 DDD	10	4017		2.25 (1.21 to 4.17)	1.54 (0.83 to 2.86)	0.17
>0.5 DDD/≤1.0 DDD	20	13 197		1.51 (0.99 to 2.29)	1.01 (0.65 to 1.57)	0.95
>1.0 DDD	13	8418		1.40 (0.82 to 2.38)	1.10 (0.65 to 1.87)	0.72
Test for trend§	-	-		-	-	0.25
**Antidepressant class by time since starting and stopping treatment**						
No current or recent use	528	512 603		1.00	1.00	
TCAs:						
First 28 days	14	5474		2.42 (1.35 to 4.37)	1.72 (0.95 to 3.10)	0.07
29-84 days after starting	16	5393		2.58 (1.56 to 4.26)	1.79 (1.08 to 2.97)	0.02
≥85 days after starting	43	18 843		2.23 (1.64 to 3.02)	1.22 (0.90 to 1.67)	0.20
1-28 days after stopping	7	3619		1.78 (0.85 to 3.72)	1.37 (0.65 to 2.89)	0.40
29-84 days after stopping	10	7040		1.35 (0.72 to 2.53)	1.04 (0.56 to 1.95)	0.90
85-182 days after stopping	24	10 726		2.31 (1.54 to 3.47)	1.82 (1.21 to 2.74)	0.004
SSRIs:						
First 28 days	32	20 688		1.50 (0.96 to 2.36)	1.41 (0.89 to 2.23)	0.14
29-84 days after starting	34	27 938		1.04 (0.70 to 1.54)	1.00 (0.67 to 1.50)	0.99
≥85 days after starting	183	127 522		1.46 (1.22 to 1.74)	1.10 (0.92 to 1.32)	0.30
1-28 days after stopping	22	15 737		1.36 (0.87 to 2.11)	1.43 (0.91 to 2.24)	0.12
29-84 days after stopping	38	30 508		1.21 (0.87 to 1.68)	1.32 (0.95 to 1.85)	0.10
85-182 days after stopping	55	46 983		1.30 (0.98 to 1.74)	1.35 (1.01 to 1.81)	0.04
Others:						
First 28 days	10	2781		3.71 (2.04 to 6.75)	2.72 (1.45 to 5.08)	0.002
29-84 days after starting	7	3505		1.84 (0.88 to 3.84)	1.48 (0.70 to 3.10)	0.30
≥85 days after starting	27	16 854		1.64 (1.13 to 2.39)	1.07 (0.72 to 1.58)	0.74
1-28 days after stopping	4	1574		2.40 (0.90 to 6.37)	2.15 (0.81 to 5.70)	0.13
29-84 days after stopping	2	3024		0.64 (0.16 to 2.53)	0.58 (0.15 to 2.28)	0.43
85-182 days after stopping	7	4542		1.76 (0.88 to 3.52)	1.43 (0.68 to 3.00)	0.35

DDD=defined daily dose; SSRI=selective serotonin reuptake inhibitor; TCA=tricyclic and related antidepressant.

*Based on numbers in adjusted analysis.

†Adjusted for age, sex, year of diagnosis of depression, severity of depression, deprivation, smoking status, alcohol intake, ethnic group (white/not recorded or non-white), coronary heart disease, diabetes, hypertension, cancer, epilepsy/seizures, hypothyroidism, osteoarthritis, asthma/chronic obstructive pulmonary disease, rheumatoid arthritis, osteoporosis, liver disease, renal disease, obsessive-compulsive disorder, statins, non-steroidal anti-inflammatory drugs, aspirin, antihypertensives, anticonvulsants, hypnotics/anxiolytics, oral contraceptives, hormone replacement therapy, antipsychotics, bisphosphonates, anticoagulants.

‡Daily doses could not be evaluated for some prescriptions.

§Test for trend uses continuous values of dose.

In the adjusted analysis of individual antidepressant drugs, we found no significant associations for any of the drugs (fig 3).

**Figure f3:**
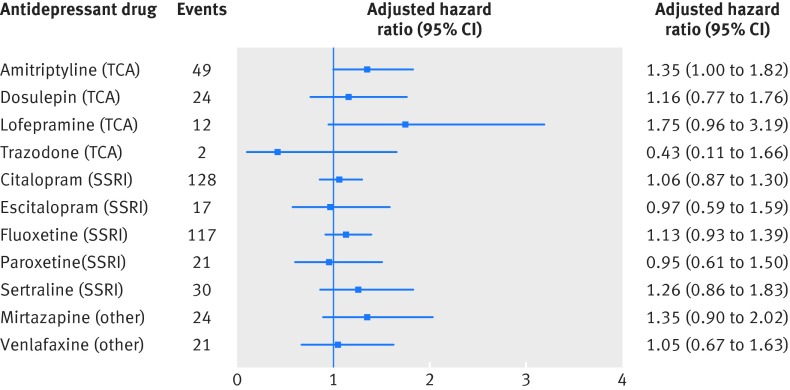
**Fig 3** Adjusted hazard ratios (compared with periods of non-use of antidepressants) for stroke or transient ischaemic attack for individual antidepressant drugs over 5 years’ follow-up. SSRI=selective serotonin reuptake inhibitor; TCA=tricyclic and related antidepressant

Adjusted hazard ratios were similar when patients who had not received any prescriptions for antidepressants during follow-up were removed (supplementary table J) and when the entire follow-up period was used (supplementary table K), but they tended to be lower when just the first year of follow-up was used in the analysis (table 4). We saw some indication that hazard rates were not proportional over the five years of follow- up, with higher hazard ratios in the later periods of follow-up for tricyclic and related antidepressants and selective serotonin reuptake inhibitors (supplementary tables F and G).

### Additional analyses

The results of analyses including confounding variables in blocks are shown in supplementary tables L to N, showing that adjustment for age, sex, deprivation, ethnic group, and year of diagnosis had a marked effect on hazard ratios, but additional adjustment for further blocks of variables had a relatively small effect. Results were similar to those of our main models which used robust standard errors when the Cox models were stratified by general practice.

### Absolute risks

Table 7 shows absolute risks of the three outcomes over one year by antidepressant class and for the individual drugs. Absolute risks of arrhythmia and myocardial infarction were highest for lofepramine (30 per 10 000 and 31 per 10 000, respectively), and for stroke/transient ischaemic attack they were highest for mirtazapine (24 per 10 000). However, the 95% confidence intervals for these values were wide and mainly overlapped with the other drugs.

**Table 7 tbl7:** Absolute risks of arrhythmia, myocardial infarction, and stroke or transient ischaemic attack over 1 year by antidepressant class and for individual drugs.

Treatment	Absolute risk per 10,000 over 1 year (95% CI)		
	Arrhythmia*	Myocardial infarction†	Stroke/TIA‡
No treatment	14 (11 to 17)	10 (8 to 12)	13 (11 to 16)
**Antidepressant class**			
TCAs	16 (11 to 23)	11 (7 to 17)	13 (9 to 19)
SSRIs	12 (9 to 16)	6 (4 to 8)	11 (8 to 14)
Other antidepressants	19 (12 to 30)	8 (4 to 16)	15 (9 to 25)
Combined antidepressants	48 (17 to 133)	17 (4 to 66)	9 (1 to 64)
**Antidepressant drug**			
TCAs:			
Amitriptyline	16 (10 to 27)	8 (4 to 16)	13 (8 to 22)
Dosulepin	10 (5 to 21)	11 (5 to 22)	15 (8 to 26)
Lofepramine	30 (15 to 60)	31 (15 to 62)	15 (6 to 40)
Trazodone	24 (7 to 78)	7 (1 to 52)	7 (1 to 48)
SSRIs:			
Citalopram	11 (8 to 15)	6 (4 to 9)	10 (7 to 14)
Escitalopram	14 (7 to 30)	7 (2 to 18)	8 (3 to 20)
Fluoxetine	11 (8 to 16)	4 (3 to 7)	14 (10 to 19)
Paroxetine	15 (9 to 28)	4 (1 to 12)	8 (4 to 18)
Sertraline	17 (10 to 29)	12 (6 to 22)	8 (4 to 18)
Others:			
Mirtazapine	17 (8 to 35)	9 (4 to 22)	24 (13 to 44)
Venlafaxine	23 (12 to 43)	9 (3 to 24)	7 (2 to 20)
All other antidepressants	13 (4 to 38)	5 (1 to 33)	8 (2 to 34)

SSRI=selective serotonin reuptake inhibitor; TCA=tricyclic and related antidepressant; TIA=transient ischaemic attack.

*Absolute risks are adjusted for confounders listed in table 2.

†Absolute risks are adjusted for confounders listed in table 5.

‡Absolute risks are adjusted for confounders listed in table 6.

## Discussion

The main findings of this large population based cohort study were that selective serotonin reuptake inhibitors were not associated with an increased risk of arrhythmia, myocardial infarction, or stroke or transient ischaemic attack in a general population cohort of people with depression aged 20 to 64 and that risk of arrhythmia was not significantly increased in patients treated with citalopram even at high doses (40 mg/day and over), although numbers in this category were relatively small. We found some evidence that selective serotonin reuptake inhibitors were associated with a reduced risk of arrhythmia and myocardial infarction. Fluoxetine was associated with the lowest risks of these two outcomes, but overall no significant differences were seen between the selective serotonin reuptake inhibitors. The risk of arrhythmia was significantly increased in the first four weeks of starting tricyclic and related antidepressants, and the tricyclic drug lofepramine was associated with a significantly increased risk of myocardial infarction in the first year of follow-up.

### Strengths and limitations of study

This study included a large representative sample of people aged 20 to 64 diagnosed as having depression in the general UK population and had a long follow-up period. All eligible patients were included, so no bias due to non-response was present, and no recall bias occurred because data on prescriptions and confounding variables were recorded prospectively before the outcomes occurred. We reduced indication bias by restricting our cohort to include only patients with a diagnosis of depression, as depression itself is an established risk factor for cardiovascular outcomes,^30^ ^31^ and separating the effects of antidepressant treatment from those of depression would otherwise be difficult. This means that our findings can be generalised only to people diagnosed as having depression.

Some bias may remain in comparisons between antidepressant drugs if the selection of a particular antidepressant was influenced by risk factors for the outcome, but we accounted for a large number of potential confounding variables in the analysis to reduce differences between comparison groups. The increased risk for lofepramine in some analyses may nevertheless reflect preferential selection of this drug in patients considered to be more prone to arrhythmias or heart disease, as this drug is viewed as being safer in overdose and less cardiotoxic than other tricyclic and related antidepressants.^32^ ^33^ The increased risk of arrhythmia for low doses of lofepramine but not higher doses supports this, whereby patients at highest risk are treated with lower doses, although numbers of events were small in both dose categories. However, in a comparison of baseline characteristics of patients who received prescriptions for different antidepressants, we saw no indication that lofepramine was prescribed more frequently than other tricyclic antidepressants to patients with cardiovascular risk factors.^22^ For example, among patients whose first antidepressant prescription was for lofepramine, 1.1% had coronary heart disease compared with 2.1% for amitriptyline, and 0.8% had a previous stroke recorded compared with 1.0% for amitriptyline. Similarly, we observed no indication that fluoxetine was prescribed more frequently than other selective serotonin reuptake inhibitors to younger patients or patients with fewer cardiovascular risk factors. For example, the mean age of patients when first treated with fluoxetine was 38.8 years, compared with 39.8 for citalopram and 38.3 for paroxetine, and the proportion of patients with hypertension when first treated with fluoxetine was 6.7%, whereas for paroxetine it was 5.3%.^22^

Some residual confounding may still be present owing to variables that either were not recorded on the database, such as dietary factors and physical activity, or were not recorded in sufficient detail for their confounding effect to be completely removed by analysis. Although we adjusted for severity of depression, this was based on a basic classification of diagnostic Read codes for depression, as depression severity scores are not routinely recorded in general practice. Numbers of patients in the different non-white ethnic groups were small, so we combined these for inclusion in the analysis, which may contribute to residual confounding. Some misclassification of the antidepressant exposure variables will have occurred, as some patients may not have taken their prescribed antidepressant or may not have taken it at the prescribed dose. This misclassification could underestimate associations with drug use. Furthermore, although the cohort was large, the number of events was small for some of the antidepressant exposure categories. In particular, there were relatively few prescriptions for citalopram at doses of 40 mg/day or more (19% of citalopram prescriptions), and only 28 diagnoses of arrhythmia in this category, so the 95% confidence interval for risk of arrhythmia with high doses of citalopram is wide, and increases in risk of up to 71% cannot be excluded.

The outcomes were not formally adjudicated in this study, but validation studies in other UK primary care databases have shown high levels of validity across a range of diseases, and we would expect levels of validity to be similar in QResearch.^34^ ^35^ For example, Khan reported high positive predictive values in validation studies of acute myocardial infarction and cerebrovascular disease.^35^ A study validating diagnostic codes for ventricular arrhythmia and sudden cardiac death reported a positive predictive value of 93%.^36^ We included information from death certificates to identify additional patients with the outcomes, which will have increased ascertainment and reduced misclassification.

### Comparison with other studies

Our results for arrhythmia are consistent with those of two other large cohort studies in finding no increased risk for citalopram,^18^ ^19^ even at high doses, and our rates of arrhythmia are of the same order of magnitude. Our study adds new information on risks associated with other antidepressant drugs and on effects of duration of treatment. Our findings contrast to some extent with those of studies that have found QT interval prolongation in patients taking citalopram.^14^ ^15^ ^16^ One cross sectional study,^15^ which included 38 397 patients aged 18 and over with an electrocardiogram recorded after prescription of antidepressant or methadone, found that QT prolongation was associated with dose for citalopram, escitalopram, and amitriptyline but not for other antidepressants examined. A study of psychiatric inpatients aged 18 and over found that most people with QT prolongation had two or more risk factors for QT prolongation, such as hypokalaemia, HIV infection, abnormal T wave morphology, and alcohol or drug use disorders, and that citalopram (including escitalopram) was significantly associated with QT prolongation after adjustment for these factors.^16^ This lack of coherence may reflect the smaller numbers of arrhythmia outcomes in the cohort studies when split by antidepressant drug and dose. Thus, power to detect an increased risk among higher antidepressant dose categories is low in comparison with studies that measure QT interval in adults receiving different doses of antidepressants and treat it as a continuous outcome variable in the analyses.^14^ ^15^ Torsades de pointes, which is the type of arrhythmia most closely related to QT interval prolongation, is extremely rare, so cohort studies (including ours) cannot rule out an association for this particular type of arrhythmia. Furthermore, a surrogate measure such as QT interval may not necessarily translate into an effect on a clinically important outcome such as arrhythmia. Our findings of an increased risk of arrhythmia in the first four weeks of starting a tricyclic antidepressant is consistent with several potential arrhythmias that can occur with tricyclic overdose in people with previously unsuspected cardiac abnormalities such as bundle branch block^37^ ^38^; our findings are important, as few studies have examined this for prescribed doses of tricyclic antidepressants.

In our previous study of antidepressants in people aged 65 and over with depression,^10^ ^25^ we found a significantly increased risk of myocardial infarction with selective serotonin reuptake inhibitors but not with tricyclic or other antidepressants. Other observational studies have found similar results for selective serotonin reuptake inhibitors,^39^ ^40^ whereas several have found no association^11^ ^12^ ^41^ ^42^ or a reduced risk^13^ ^43^ ^44^; few studies have assessed risks for individual antidepressants. A meta-analysis of 16 observational studies concluded that use of neither selective serotonin reuptake inhibitors nor tricyclic antidepressants is associated with an increased risk of coronary heart disease,^45^ but only two studies were restricted to patients with depression. These contradictory findings are likely to be due to differences between studies, as they vary considerably in their sizes and inclusion criteria. Several studies either did not restrict their study sample to patients with depression or did not account for depression in the analysis and so are highly susceptible to indication bias because depression is a strong risk factor for cardiovascular disease^11^ ^12^ ^13^; some studies are only in older or postmenopausal populations^10^ ^39^ ^42^; and one was an interview based case-control study prone to recall bias.^44^ Why our results differ from those of our previous study in older people, which had a very similar study design, is unclear,^10^ but it could be due to the larger number of myocardial infarction events (n=2350) in the older cohort or increased susceptibility to side effects in older people resulting from age related pharmacokinetic changes,^46^ or the high prevalence of multimorbidity and use of concomitant drugs in older people may result in interactions giving different patterns of risk with antidepressant use.

Observational studies of antidepressants and stroke have shown a more consistent pattern; several studies have found an increased risk of stroke with selective serotonin reuptake inhibitor use.^10^ ^42^ ^47^ ^48^ ^49^ A systematic review and meta-analysis of 13 observational studies of selective serotonin reuptake inhibitors and stroke reported that selective serotonin reuptake inhibitors were associated with an increased risk of all types of stroke (overall adjusted odds ratio 1.40, 95% confidence interval 1.09 to 1.80) and that the risk was still significantly increased when the analysis was restricted to the studies in which potential confounding by depression was considered.^9^ In a subgroup analysis by age group, the combined odds ratio for all types of stroke associated with selective serotonin reuptake inhibitor use was significant only in the four studies restricted to people aged at least 50 years (overall adjusted odds ratio 1.58, 1.06 to 2.36),^10^ ^42^ ^50^ ^51^ and no significantly increased risk was seen in studies with no age restriction (overall adjusted odds ratio 1.13, 0.91 to 1.39). This concurs with our findings in this study of no association between selective serotonin reuptake inhibitors and stroke in people aged 20 to 64 and of an increased risk in our previous study in people aged 65 and over.^10^

### Clinical implications and future research

Prescription of antidepressants is a complex process, involving balancing of risks and benefits for different antidepressants and doses, accounting for severity of depression, and considering patients’ risk factors, comorbidities, and preferences. The results of this study in adults aged 20 to 64 are reassuring in light of recent concerns about citalopram and potential risk of arrhythmia; however, as only small numbers of patients were treated with high doses of citalopram, we cannot rule out the possibility of an increased risk. We suggest that high doses of citalopram should not be prescribed without a strong indication, particularly in patients with any risk factors for an increased QT interval. We also found no evidence that selective serotonin reuptake inhibitors are associated with an increased risk of myocardial infarction or stroke/transient ischaemic attack in this age group; they may even be associated with a reduced risk of myocardial infarction and arrhythmia, particularly for fluoxetine. The potential cardioprotective effects of selective serotonin reuptake inhibitors, particularly fluoxetine, warrant further investigation.

The risk of arrhythmia was increased during the first 28 days of taking tricyclic and related antidepressants, and among the antidepressants studied lofepramine had the highest risks of arrhythmia, myocardial infarction, and stroke/transient ischaemic attack. This finding may reflect selective prescribing of lofepramine, as it is generally considered to be safer than other tricyclic and related antidepressants in overdose, but could also indicate increased risks when it is taken at doses typically prescribed in primary care. Further research using other designs such as the self controlled case series approach may help to elucidate this association.

### Conclusions

This large observational study has found no evidence that selective serotonin reuptake inhibitors are associated with an increased risk of arrhythmia, myocardial infarction, or stroke/transient ischaemic attack in people with depression aged 20 to 64, but some indication that they are associated with a reduced risk of myocardial infarction and arrhythmia, particularly for fluoxetine. Citalopram was not significantly associated with an increased risk of arrhythmia, even at higher doses, although the confidence interval was wide. These findings are reassuring in light of recent safety concerns about selective serotonin reuptake inhibitors.

**What is already known on this topic**

Depression is a common condition, and antidepressants—particularly selective serotonin reuptake inhibitors—are increasingly used in its treatment
Rates of cardiovascular disease are higher in people with depression, but whether different antidepressant treatments increase or reduce these rates is unclear
High doses of certain antidepressants, including citalopram, can cause QT prolongation, which may increase the risk of arrhythmia, but this is not established

**What this study adds**

This study found no evidence that selective serotonin reuptake inhibitors as a class are associated with an increased risk of arrhythmia and stroke or transient ischaemic attack in people with depression aged 20 to 64
No evidence was found that citalopram is associated with a significantly increased risk of arrhythmia, even at high doses
Some indication was seen of a reduced risk of myocardial infarction for selective serotonin reuptake inhibitors, particularly fluoxetine

## Web Extra

Extra material supplied by the author

## Notes

We acknowledge the contribution of practices that contribute to the QResearch, as well as Egton Medical Information Systems (EMIS) and the University of Nottingham for expertise in establishing, developing, and supporting the database. We acknowledge the Office of National Statistics for providing mortality data.

Ethical approval: The project has been independently peer reviewed and accepted by the QResearch Scientific board and has been approved in accordance with the agreed procedure with the Trent Research Ethics Committee (reference number: MREC/03/4/021).

Transparency declaration: The lead author (the manuscript’s guarantor) affirms that this manuscript is an honest, accurate, and transparent account of the study being reported; that no important aspects of the study have been omitted; and that any discrepancies from the study as planned (and, if relevant, registered) have been explained.

Data sharing: The patient level data from the QResearch are specifically licensed according to its governance framework. See www.qresearch.org for further details.
