# Supplementary material for: Antidepressant use and risk of cardiovascular outcomes in people aged 20 to 64: cohort study using primary care database
Source: BMJ. 2016 Mar 22;352:i1350. doi: 10.1136/bmj.i1350 (PMC4804126; doi:10.1136/bmj.i1350)
Supplement: Supplementary file 1 — Appendix [file couc027737.ww1_default.pdf]

Outcome	Code type	Code	Code Description
Arrhythmia	Read code	327Z	ECG: supraventric. arryth. NOS
Arrhythmia	Read code	328Z	ECG: ventricular arrhythmia NOS
Arrhythmia	Read code	EMISNQAS1	Asystolic vasovagal syncope
Arrhythmia	Read code	F2560	Hypsarrhythmia
Arrhythmia	Read code	G57-1	Cardiac arrhythmias
Arrhythmia	Read code	G570	Paroxysmal supraventricular tachycardia
Arrhythmia	Read code	G570-99	Parox. supravent. tachycardia
Arrhythmia	Read code	G5700	Paroxysmal atrial tachycardia
Arrhythmia	Read code	G5701	Paroxysmal atrioventricular tachycardia
Arrhythmia	Read code	G5702	Paroxysmal junctional tachycardia
Arrhythmia	Read code	G5703	Paroxysmal nodal tachycardia
Arrhythmia	Read code	G570z	Paroxysmal supraventricular tachycardia NOS
Arrhythmia	Read code	G571	Paroxysmal ventricular tachycardia
Arrhythmia	Read code	G571-1	Ventricular tachycardia
Arrhythmia	Read code	G571-99	Paroxysmal ventric. tachyc.
Arrhythmia	Read code	G572	Paroxysmal tachycardia unspecified
Arrhythmia	Read code	G5720	Essential paroxysmal tachycardia
Arrhythmia	Read code	G5721	Bouveret-Hoffmann syndrome
Arrhythmia	Read code	G572z	Paroxysmal tachycardia NOS
Arrhythmia	Read code	G573	Atrial fibrillation and flutter
Arrhythmia	Read code	G5730	Atrial fibrillation
Arrhythmia	Read code	G5731	Atrial flutter
Arrhythmia	Read code	G5732	Paroxysmal atrial fibrillation
Arrhythmia	Read code	G5733	Non-rheumatic atrial fibrillation
Arrhythmia	Read code	G573z	Atrial fibrillation and flutter NOS
Arrhythmia	Read code	G574	Ventricular fibrillation and flutter
Arrhythmia	Read code	G5740	Ventricular fibrillation
Arrhythmia	Read code	G5740-1	Cardiac arrest-ventricular fibrillation
Arrhythmia	Read code	G5741	Ventricular flutter
Arrhythmia	Read code	G574z	Ventricular fibrillation and flutter NOS
Arrhythmia	Read code	G576-1	Premature beats
Arrhythmia	Read code	G5760	Ectopic beats unspecified

Outcome	Code type	Code	Code Description
Arrhythmia	Read code	G5760-1	Extrasystoles
Arrhythmia	Read code	G5761	Supraventricular ectopic beats
Arrhythmia	Read code	G5762	Ventricular ectopic beats
Arrhythmia	Read code	G5763	Atrial premature depolarization
Arrhythmia	Read code	G5764	Junctional premature depolarization
Arrhythmia	Read code	G5765	Ventricular premature depolarization
Arrhythmia	Read code	G576z	Ectopic beats NOS
Arrhythmia	Read code	G577	Sinus arrhythmia
Arrhythmia	Read code	G57y	Other cardiac dysrhythmias
Arrhythmia	Read code	G57y-99	Cardiac dysrhythmias NOS
Arrhythmia	Read code	G57y0	Persistent sinus bradycardia
Arrhythmia	Read code	G57y1	Severe sinus bradycardia
Arrhythmia	Read code	G57y3	Sick sinus syndrome
Arrhythmia	Read code	G57y4	Sinoatrial node dysfunction NOS
Arrhythmia	Read code	G57y5	Wandering atrial pacemaker
Arrhythmia	Read code	G57y6	Nodal rhythm disorder
Arrhythmia	Read code	G57y7	Sinus tachycardia
Arrhythmia	Read code	G57y8	Bigeminal pulse
Arrhythmia	Read code	G57y9	Supraventricular tachycardia NOS
Arrhythmia	Read code	G57yA	Re-entry ventricular arrhythmia
Arrhythmia	Read code	G57yz	Other cardiac dysrhythmia NOS
Arrhythmia	Read code	G57z	Cardiac dysrhythmia NOS
Arrhythmia	Read code	G57z-99	Cardiac dysrhythmias NOS
Arrhythmia	Read code	Gyu5a	[X]Other specified cardiac arrhythmias
Arrhythmia	ICD9	427	Cardiac dysrhythmias
Arrhythmia	ICD9	427	Paroxysmal supraventricular tachycardia
Arrhythmia	ICD9	427.1	Paroxysmal ventricular tachycardia
Arrhythmia	ICD9	427.2	Paroxysmal tachycardia, unspecified
Arrhythmia	ICD9	427.3	Atrial fibrillation and flutter
Arrhythmia	ICD9	427.31	Atrial fibrillation
Arrhythmia	ICD9	427.32	Atrial flutter

Outcome	Code type	Code	Code Description
Arrhythmia	ICD9	427.4	Ventricular fibrillation and flutter
Arrhythmia	ICD9	427.41	Ventricular fibrillation
Arrhythmia	ICD9	427.42	Ventricular flutter
Arrhythmia	ICD9	427.6	Premature beats
Arrhythmia	ICD9	427.6	Premature beats, unspecified
Arrhythmia	ICD9	427.61	Supraventricular premature beats
Arrhythmia	ICD9	427.69	Other
Arrhythmia	ICD9	427.8	Other specified cardiac dysrhythmias
Arrhythmia	ICD9	427.81	Sinoatrial node dysfunction
Arrhythmia	ICD9	427.89	Other
Arrhythmia	ICD9	427.9	Cardiac dysrhythmia, unspecified
Arrhythmia	ICD10	I47	Paroxysmal tachycardia
Arrhythmia	ICD10	I47.0	Re-entry ventricular arrhythmia
Arrhythmia	ICD10	I47.1	Supraventricular tachycardia
Arrhythmia	ICD10	I47.2	Ventricular tachycardia
Arrhythmia	ICD10	I47.9	Paroxysmal tachycardia, unspecified
Arrhythmia	ICD10	I48	Atrial fibrillation and flutter
Arrhythmia	ICD10	I49	Other cardiac arrhythmias
Arrhythmia	ICD10	I49.0	Ventricular fibrillation and flutter
Arrhythmia	ICD10	I49.1	Atrial premature depolarization
Arrhythmia	ICD10	I49.2	Junctional premature depolarization
Arrhythmia	ICD10	I49.3	Ventricular premature depolarization
Arrhythmia	ICD10	I49.4	Other and unspecified premature depolarization
Arrhythmia	ICD10	I49.5	Sick sinus syndrome
Arrhythmia	ICD10	I49.8	Other specified cardiac arrhythmias
Arrhythmia	ICD10	I49.9	Cardiac arrhythmia, unspecified
Myocardial infarction	Read code	EMISR4QFI1	First myocardial infarction
Myocardial infarction	Read code	G30	Acute myocardial infarction
Myocardial infarction	Read code	G30-1	Attack - heart
Myocardial infarction	Read code	G30-2	Coronary thrombosis

Outcome	Code type	Code	Code Description
Myocardial infarction	Read code	G30-3	Cardiac rupture following myocardial infarction (MI)
Myocardial infarction	Read code	G30-4	Heart attack
Myocardial infarction	Read code	G30-5	MI - acute myocardial infarction
Myocardial infarction	Read code	G30-6	Thrombosis - coronary
Myocardial infarction	Read code	G30-7	Silent myocardial infarction
Myocardial infarction	Read code	G30-98	Coronary thrombosis
Myocardial infarction	Read code	G30-99	Myocardial Infarction
Myocardial infarction	Read code	G300	Acute anterolateral infarction
Myocardial infarction	Read code	G301	Other specified anterior myocardial infarction
Myocardial infarction	Read code	G3010	Acute anteroapical infarction
Myocardial infarction	Read code	G3011	Acute anteroseptal infarction
Myocardial infarction	Read code	G301z	Anterior myocardial infarction NOS
Myocardial infarction	Read code	G302	Acute inferolateral infarction
Myocardial infarction	Read code	G303	Acute inferoposterior infarction
Myocardial infarction	Read code	G304	Posterior myocardial infarction NOS
Myocardial infarction	Read code	G305	Lateral myocardial infarction NOS
Myocardial infarction	Read code	G306	True posterior myocardial infarction
Myocardial infarction	Read code	G307	Acute subendocardial infarction
Myocardial infarction	Read code	G3070	Acute non-Q wave infarction
Myocardial infarction	Read code	G3071	Acute non-ST segment elevation myocardial infarction
Myocardial infarction	Read code	G308	Inferior myocardial infarction NOS
Myocardial infarction	Read code	G309	Acute Q-wave infarct
Myocardial infarction	Read code	G30A	Mural thrombosis
Myocardial infarction	Read code	G30B	Acute posterolateral myocardial infarction
Myocardial infarction	Read code	G30X	Acute transmural myocardial infarction of unspecif site
Myocardial infarction	Read code	G30X0	Acute ST segment elevation myocardial infarction
Myocardial infarction	Read code	G30y	Other acute myocardial infarction
Myocardial infarction	Read code	G30y0	Acute atrial infarction
Myocardial infarction	Read code	G30y1	Acute papillary muscle infarction
Myocardial infarction	Read code	G30y2	Acute septal infarction
Myocardial infarction	Read code	G30yz	Other acute myocardial infarction NOS
Myocardial infarction	Read code	G30z	Acute myocardial infarction NOS

Outcome	Code type	Code	Code Description
Myocardial infarction	Read code	G310-1	Dressler's syndrome
Myocardial infarction	Read code	G31y1	Microinfarction of heart
Myocardial infarction	Read code	G35	Subsequent myocardial infarction
Myocardial infarction	Read code	G350	Subsequent myocardial infarction of anterior wall
Myocardial infarction	Read code	G351	Subsequent myocardial infarction of inferior wall
Myocardial infarction	Read code	G353	Subsequent myocardial infarction of other sites
Myocardial infarction	Read code	G35X	Subsequent myocardial infarction of unspecified site
Myocardial infarction	Read code	G360	Haemopericardium/current comp folow acut myocard infarct
Myocardial infarction	Read code	G361	Atrial septal defect/curr comp folow acut myocardal infarct
Myocardial infarction	Read code	G362	Ventric septal defect/curr comp fol acut myocardal infarctn
Myocardial infarction	Read code	G363	Ruptur cardiac wall w'out haemopericard/cur comp fol ac MI
Myocardial infarction	Read code	G364	Ruptur chordae tendinae/curr comp fol acute myocard infarct
Myocardial infarction	Read code	G365	Rupture papillary muscle/curr comp fol acute myocard infarct
Myocardial infarction	Read code	G366	Thrombosis atrium,auric append&vent/curr comp foll acute MI
Myocardial infarction	Read code	G38	Postoperative myocardial infarction
Myocardial infarction	Read code	G380	Postoperative transmural myocardial infarction anterior wall
Myocardial infarction	Read code	G381	Postoperative transmural myocardial infarction inferior wall
Myocardial infarction	Read code	G382	Postoperative transmural myocardial infarction other sites
Myocardial infarction	Read code	G383	Postoperative transmural myocardial infarction unspec site
Myocardial infarction	Read code	G384	Postoperative subendocardial myocardial infarction
Myocardial infarction	Read code	G38z	Postoperative myocardial infarction, unspecified
Myocardial infarction	Read code	G501	Post infarction pericarditis
Myocardial infarction	Read code	Gyu34	[X]Acute transmural myocardial infarction of unspecif site
Myocardial infarction	ICD9	410	Acute myocardial infarction
Myocardial infarction	ICD9	410	Of anterolateral wall
Myocardial infarction	ICD9	410.1	Of other anterior wall
Myocardial infarction	ICD9	410.2	Of inferolateral wall
Myocardial infarction	ICD9	410.3	Of inferoposterior wall
Myocardial infarction	ICD9	410.4	Of other inferior wall
Myocardial infarction	ICD9	410.5	Of other lateral wall
Myocardial infarction	ICD9	410.6	True posterior wall infarction

Outcome	Code type	Code	Code Description
Myocardial infarction	ICD9	410.7	Subendocardial infarction
Myocardial infarction	ICD9	410.8	Of other specified sites
Myocardial infarction	ICD9	410.9	Unspecified site
Myocardial infarction	ICD10	I21	Acute myocardial infarction
Myocardial infarction	ICD10	I21.0	Acute transmural myocardial infarction of anterior wall
Myocardial infarction	ICD10	I21.1	Acute transmural myocardial infarction of inferior wall
Myocardial infarction	ICD10	I21.2	Acute transmural myocardial infarction of other sites
Myocardial infarction	ICD10	I21.3	Acute transmural myocardial infarction of unspecified site
Myocardial infarction	ICD10	I21.4	Acute subendocardial myocardial infarction
Myocardial infarction	ICD10	I21.9	Acute myocardial infarction, unspecified
Myocardial infarction	ICD10	I22	Subsequent myocardial infarction
Myocardial infarction	ICD10	I22.0	Subsequent myocardial infarction of anterior wall
Myocardial infarction	ICD10	I22.1	Subsequent myocardial infarction of inferior wall
Myocardial infarction	ICD10	I22.8	Subsequent myocardial infarction of other sites
Myocardial infarction	ICD10	I22.9	Subsequent myocardial infarction of unspecified site
Stroke or TIA	Read code	F4236	Amaurosis fugax
Stroke or TIA	Read code	Fyu55	[X]Other transnt cerebral ischaemic attacks+related syndroms
Stroke or TIA	Read code	G61	Intracerebral haemorrhage
Stroke or TIA	Read code	G61-1	CVA - cerebrovascular accid due to intracerebral haemorrhage
Stroke or TIA	Read code	G61-2	Stroke due to intracerebral haemorrhage
Stroke or TIA	Read code	G61-98	Cerebral haemorrhage NOS
Stroke or TIA	Read code	G61-99	Cerebral haemorrhage
Stroke or TIA	Read code	G610	Cortical haemorrhage
Stroke or TIA	Read code	G611	Internal capsule haemorrhage
Stroke or TIA	Read code	G612	Basal nucleus haemorrhage
Stroke or TIA	Read code	G613	Cerebellar haemorrhage
Stroke or TIA	Read code	G614	Pontine haemorrhage
Stroke or TIA	Read code	G615	Bulbar haemorrhage
Stroke or TIA	Read code	G616	External capsule haemorrhage
Stroke or TIA	Read code	G618	Intracerebral haemorrhage, multiple localized

Outcome	Code type	Code	Code Description
Stroke or TIA	Read code	G61X	Intracerebral haemorrhage in hemisphere, unspecified
Stroke or TIA	Read code	G61X0	Left sided intracerebral haemorrhage, unspecified
Stroke or TIA	Read code	G61X1	Right sided intracerebral haemorrhage, unspecified
Stroke or TIA	Read code	G61z	Intracerebral haemorrhage NOS
Stroke or TIA	Read code	G63y0	Cerebral infarct due to thrombosis of precerebral arteries
Stroke or TIA	Read code	G63y1	Cerebral infarction due to embolism of precerebral arteries
Stroke or TIA	Read code	G64	Cerebral arterial occlusion
Stroke or TIA	Read code	G64-1	CVA - cerebral artery occlusion
Stroke or TIA	Read code	G64-2	Infarction - cerebral
Stroke or TIA	Read code	G64-3	Stroke due to cerebral arterial occlusion
Stroke or TIA	Read code	G640	Cerebral thrombosis
Stroke or TIA	Read code	G6400	Cerebral infarction due to thrombosis of cerebral arteries
Stroke or TIA	Read code	G641	Cerebral embolism
Stroke or TIA	Read code	G641-1	Cerebral embolus
Stroke or TIA	Read code	G6410	Cerebral infarction due to embolism of cerebral arteries
Stroke or TIA	Read code	G64z	Cerebral infarction NOS
Stroke or TIA	Read code	G64z-1	Brainstem infarction NOS
Stroke or TIA	Read code	G64z-2	Cerebellar infarction
Stroke or TIA	Read code	G64z-99	Cerebral A. occlusion NOS
Stroke or TIA	Read code	G64z0	Brainstem infarction
Stroke or TIA	Read code	G64z1	Wallenberg syndrome
Stroke or TIA	Read code	G64z1-1	Lateral medullary syndrome
Stroke or TIA	Read code	G64z2	Left sided cerebral infarction
Stroke or TIA	Read code	G64z3	Right sided cerebral infarction
Stroke or TIA	Read code	G64z4	Infarction of basal ganglia
Stroke or TIA	Read code	G65	Transient cerebral ischaemia
Stroke or TIA	Read code	G65-1	Drop attack
Stroke or TIA	Read code	G65-2	Transient ischaemic attack
Stroke or TIA	Read code	G65-3	Vertebro-basilar insufficiency
Stroke or TIA	Read code	G65-99	Transient Ischaemic Attacks
Stroke or TIA	Read code	G650	Basilar artery syndrome
Stroke or TIA	Read code	G650-1	Insufficiency - basilar artery

Outcome	Code type	Code	Code Description
Stroke or TIA	Read code	G651	Vertebral artery syndrome
Stroke or TIA	Read code	G6510	Vertebro-basilar artery syndrome
Stroke or TIA	Read code	G652	Subclavian steal syndrome
Stroke or TIA	Read code	G653	Carotid artery syndrome hemispheric
Stroke or TIA	Read code	G654	Multiple and bilateral precerebral artery syndromes
Stroke or TIA	Read code	G656	Vertebrobasilar insufficiency
Stroke or TIA	Read code	G65y	Other transient cerebral ischaemia
Stroke or TIA	Read code	G65z	Transient cerebral ischaemia NOS
Stroke or TIA	Read code	G65z-99	Transient Ischaemic Attacks
Stroke or TIA	Read code	G65z0	Impending cerebral ischaemia
Stroke or TIA	Read code	G65z1	Intermittent cerebral ischaemia
Stroke or TIA	Read code	G65zz	Transient cerebral ischaemia NOS
Stroke or TIA	Read code	G66	Stroke and cerebrovascular accident unspecified
Stroke or TIA	Read code	G66-1	CVA unspecified
Stroke or TIA	Read code	G66-2	Stroke unspecified
Stroke or TIA	Read code	G66-3	CVA - Cerebrovascular accident unspecified
Stroke or TIA	Read code	G66-98	Stroke/CVA - undefined
Stroke or TIA	Read code	G66-99	Stroke
Stroke or TIA	Read code	G660	Middle cerebral artery syndrome
Stroke or TIA	Read code	G661	Anterior cerebral artery syndrome
Stroke or TIA	Read code	G662	Posterior cerebral artery syndrome
Stroke or TIA	Read code	G663	Brain stem stroke syndrome
Stroke or TIA	Read code	G664	Cerebellar stroke syndrome
Stroke or TIA	Read code	G665	Pure motor lacunar syndrome
Stroke or TIA	Read code	G666	Pure sensory lacunar syndrome
Stroke or TIA	Read code	G667	Left sided CVA
Stroke or TIA	Read code	G668	Right sided CVA
Stroke or TIA	Read code	G6760	Cereb infarct due cerebral venous thrombosis, nonpyogenic
Stroke or TIA	Read code	G6W	Cereb infarct due unsp occlus/stenos precerebr arteries
Stroke or TIA	Read code	G6X	Cerebrl infarctn due/unspcf occlusn or sten/cerebrl artrs
Stroke or TIA	Read code	Gyu62	[X]Other intracerebral haemorrhage
Stroke or TIA	Read code	Gyu63	[X]Cerebrl infarctn due/unspcf occlusn or sten/cerebrl artrs

Outcome	Code type	Code	Code Description
Stroke or TIA	Read code	Gyu64	[X]Other cerebral infarction
Stroke or TIA	Read code	Gyu65	[X]Occlusion and stenosis of other precerebral arteries
Stroke or TIA	Read code	Gyu66	[X]Occlusion and stenosis of other cerebral arteries
Stroke or TIA	Read code	Gyu6F	[X]Intracerebral haemorrhage in hemisphere, unspecified
Stroke or TIA	Read code	Gyu6G	[X]Cereb infarct due unsp occlus/stenos precerebr arteries
Stroke or TIA	Read code	ZV12D	[V]Personal history of transient ischaemic attack
Stroke or TIA	ICD9	430	Subarachnoid hemorrhage
Stroke or TIA	ICD9	431	Intracerebral hemorrhage
Stroke or TIA	ICD9	432	Other and unspecified intracranial hemorrhage
Stroke or TIA	ICD9	432	Nontraumatic extradural hemorrhage
Stroke or TIA	ICD9	432.1	Subdural hemorrhage
Stroke or TIA	ICD9	432.9	Hemorrhage, intracranial, NOS
Stroke or TIA	ICD9	433	Occlusion and stenosis of precerebral arteries
Stroke or TIA	ICD9	433	Occlusion and stenosis of basilar artery
Stroke or TIA	ICD9	433	Occlusion and stenosis of basilar artery without cerebral infarction
Stroke or TIA	ICD9	433.01	Occlusion and stenosis of basilar artery with cerebral infarction
Stroke or TIA	ICD9	433.1	Occlusion and stenosis of carotid artery
Stroke or TIA	ICD9	433.1	Occlusion and stenosis of carotid artery without cerebral infarction
Stroke or TIA	ICD9	433.11	Occlusion and stenosis of carotid artery with cerebral infarction
Stroke or TIA	ICD9	433.2	Occlusion and stenosis of vertebral artery
Stroke or TIA	ICD9	433.2	Occlusion and stenosis of vertebral artery without cerebral infarction
Stroke or TIA	ICD9	433.21	Occlusion and stenosis of vertebral artery with cerebral infarction
Stroke or TIA	ICD9	433.3	Occlusion and stenosis of multiple and bilateral precerebral arteries
Stroke or TIA	ICD9	433.3	Occlusion and stenosis of multiple and bilateral precerebral arteries without cerebral infarction
Stroke or TIA	ICD9	433.8	Occlusion and stenosis of other specified precerebral artery
Stroke or TIA	ICD9	433.8	Occlusion and stenosis of other specified precerebral artery without cerebral infarction
Stroke or TIA	ICD9	433.81	Occlusion and stenosis of other specified precerebral artery with cerebral infarction
Stroke or TIA	ICD9	433.9	Occlusion and stenosis of unspecified precerebral artery
Stroke or TIA	ICD9	433.9	Occlusion and stenosis of unspecified precerebral artery without cerebral infarction
Stroke or TIA	ICD9	433.91	Occlusion and stenosis of unspecified precerebral artery with cerebral infarction
Stroke or TIA	ICD9	434	Occlusion of cerebral arteries

Outcome	Code type	Code	Code Description
Stroke or TIA	ICD9	434	Cerebral thrombosis
Stroke or TIA	ICD9	434	Cerebral thrombosis without cerebral infarction
Stroke or TIA	ICD9	434.01	Cerebral thrombosis with cerebral infarction
Stroke or TIA	ICD9	434.1	Cerebral embolism
Stroke or TIA	ICD9	434.1	Cerebral embolism without cerebral infarction
Stroke or TIA	ICD9	434.11	Cerebral embolism with cerebral infarction
Stroke or TIA	ICD9	434.9	Cerebral artery occlusion unspecified
Stroke or TIA	ICD9	434.9	Cerebral artery occlusion unspecified without cerebral infarction
Stroke or TIA	ICD9	434.91	Cerebral artery occlusion unspecified with cerebral infarction
Stroke or TIA	ICD9	435	Transient cerebral ischemia
Stroke or TIA	ICD9	435	Basilar artery syndrome
Stroke or TIA	ICD9	435.1	Vertebral artery syndrome
Stroke or TIA	ICD9	435.2	Subclavian steal syndrome
Stroke or TIA	ICD9	435.3	Vertebrobasilar artery syndrome
Stroke or TIA	ICD9	435.9	Transient ischemic attack, unspec.
Stroke or TIA	ICD9	436	Acute but ill-defined cerebrovascular disease
Stroke or TIA	ICD9	437	Other and ill-defined cerebrovascular disease
Stroke or TIA	ICD9	437	Cerebral atherosclerosis
Stroke or TIA	ICD9	437.1	Other generalized ischemic cerebrovascular disease
Stroke or TIA	ICD9	437.3	Cerebral aneurysm nonruptured
Stroke or TIA	ICD9	437.4	Cerebral arteritis
Stroke or TIA	ICD9	437.5	Moyamoya disease
Stroke or TIA	ICD9	437.6	Nonpyogenic thrombosis of intracranial venous sinus
Stroke or TIA	ICD9	437.7	Transient global amnesia
Stroke or TIA	ICD9	437.8	Other ill-defined cerebrovascular disease
Stroke or TIA	ICD9	437.9	Unspecified cerebrovascular disease
Stroke or TIA	ICD10	G46*	Vascular syndromes of brain in cerebrovascular diseases (I60-I67+)
Stroke or TIA	ICD10	G46.0*	Middle cerebral artery syndrome (I66.0+)
Stroke or TIA	ICD10	G46.1*	Anterior cerebral artery syndrome (I66.1+)
Stroke or TIA	ICD10	G46.2*	Posterior cerebral artery syndrome (I66.2+)
Stroke or TIA	ICD10	G46.3*	Brain stem stroke syndrome (I60 - I67+)

Outcome	Code type	Code	Code Description
Stroke or TIA	ICD10	G46.4*	Cerebellar stroke syndrome (I60 - I67+)
Stroke or TIA	ICD10	G46.5*	Pure motor lacunar syndrome (I60 - I67+)
Stroke or TIA	ICD10	G46.6*	Pure sensory lacunar syndrome (I60 - I67+)
Stroke or TIA	ICD10	G46.7*	Other lacunar syndromes (I60 - I67+)
Stroke or TIA	ICD10	G46.8*	Other vascular syndromes of brain in cerebrovascular diseases (I60 - I67+)
Stroke or TIA	ICD10	I60	Subarachnoid haemorrhage
Stroke or TIA	ICD10	I60.0	Subarachnoid haemorrhage from carotid siphon and bifurcation
Stroke or TIA	ICD10	I60.1	Subarachnoid haemorrhage from middle cerebral artery
Stroke or TIA	ICD10	I60.2	Subarachnoid haemorrhage from anterior communicating artery
Stroke or TIA	ICD10	I60.3	Subarachnoid haemorrhage from posterior communicating artery
Stroke or TIA	ICD10	I60.4	Subarachnoid haemorrhage from basilar artery
Stroke or TIA	ICD10	I60.5	Subarachnoid haemorrhage from vertebral artery
Stroke or TIA	ICD10	I60.6	Subarachnoid haemorrhage from other intracranial arteries
Stroke or TIA	ICD10	I60.7	Subarachnoid haemorrhage from intracranial artery, unspecified
Stroke or TIA	ICD10	I60.8	Other subarachnoid haemorrhage
Stroke or TIA	ICD10	I60.9	Subarachnoid haemorrhage, unspecified
Stroke or TIA	ICD10	I61	Intracerebral haemorrhage
Stroke or TIA	ICD10	I61.0	Intracerebral haemorrhage in hemisphere, subcortical
Stroke or TIA	ICD10	I61.1	Intracerebral haemorrhage in hemisphere, cortical
Stroke or TIA	ICD10	I61.2	Intracerebral haemorrhage in hemisphere, unspecified
Stroke or TIA	ICD10	I61.3	Intracerebral haemorrhage in brain stem
Stroke or TIA	ICD10	I61.4	Intracerebral haemorrhage in cerebellum
Stroke or TIA	ICD10	I61.5	Intracerebral haemorrhage, intraventricular
Stroke or TIA	ICD10	I61.6	Intracerebral haemorrhage, multiple localized
Stroke or TIA	ICD10	I61.8	Other intracerebral haemorrhage
Stroke or TIA	ICD10	I61.9	Intracerebral haemorrhage, unspecified
Stroke or TIA	ICD10	I62	Other nontraumatic intracranial haemorrhage
Stroke or TIA	ICD10	I62.0	Subdural haemorrhage (acute)(nontraumatic)
Stroke or TIA	ICD10	I62.1	Nontraumatic extradural haemorrhage
Stroke or TIA	ICD10	I62.9	Intracranial haemorrhage (nontraumatic), unspecified
Stroke or TIA	ICD10	I63	Cerebral infarction
Stroke or TIA	ICD10	I63.0	Cerebral infarction due to thrombosis of precerebral arteries

Outcome	Code type	Code	Code Description
Stroke or TIA	ICD10	I63.1	Cerebral infarction due to embolism of precerebral arteries
Stroke or TIA	ICD10	I63.2	Cerebral infarction due to unspecified occlusion or stenosis of precerebral arteries
Stroke or TIA	ICD10	I63.3	Cerebral infarction due to thrombosis of cerebral arteries
Stroke or TIA	ICD10	I63.4	Cerebral infarction due to embolism of cerebral arteries
Stroke or TIA	ICD10	I63.5	Cerebral infarction due to unspecified occlusion or stenosis of cerebral arteries
Stroke or TIA	ICD10	I63.6	Cerebral infarction due to cerebral venous thrombosis, nonpyogenic
Stroke or TIA	ICD10	I63.8	Other cerebral infarction
Stroke or TIA	ICD10	I63.9	Cerebral infarction, unspecified
Stroke or TIA	ICD10	I64	Stroke, not specified as haemorrhage or infarction
Stroke or TIA	ICD10	I65	Occlusion and stenosis of precerebral arteries, not resulting in cerebral infarction
Stroke or TIA	ICD10	I65.0	Occlusion and stenosis of vertebral artery
Stroke or TIA	ICD10	I65.1	Occlusion and stenosis of basilar artery
Stroke or TIA	ICD10	I65.2	Occlusion and stenosis of carotid artery
Stroke or TIA	ICD10	I65.3	Occlusion and stenosis of multiple and bilateral precerebral arteries
Stroke or TIA	ICD10	I65.8	Occlusion and stenosis of other precerebral artery
Stroke or TIA	ICD10	I65.9	Occlusion and stenosis of unspecified precerebral artery
Stroke or TIA	ICD10	I66	Occlusion and stenosis of cerebral arteries, not resulting in cerebral infarction
Stroke or TIA	ICD10	I66.0	Occlusion and stenosis of middle cerebral artery
Stroke or TIA	ICD10	I66.1	Occlusion and stenosis of anterior cerebral artery
Stroke or TIA	ICD10	I66.2	Occlusion and stenosis of posterior cerebral artery
Stroke or TIA	ICD10	I66.3	Occlusion and stenosis of cerebellar arteries
Stroke or TIA	ICD10	I66.4	Occlusion and stenosis of multiple and bilateral cerebral arteries
Stroke or TIA	ICD10	I66.8	Occlusion and stenosis of other cerebral artery
Stroke or TIA	ICD10	I66.9	Occlusion and stenosis of unspecified cerebral artery
Stroke or TIA	ICD10	I67	Other cerebrovascular diseases
Stroke or TIA	ICD10	I67.8	Other specified cerebrovascular diseases
Stroke or TIA	ICD10	I67.9	Cerebrovascular disease, unspecified
Stroke or TIA	ICD10	I68*	Cerebrovascular disorders in diseases classified elsewhere
Stroke or TIA	ICD10	I68.8*	Other cerebrovascular disorders in diseases classified elsewhere
Stroke or TIA	ICD10	I69	Sequelae of cerebrovascular disease
Stroke or TIA	ICD10	I69.0	Sequelae of subarachnoid haemorrhage
Stroke or TIA	ICD10	I69.1	Sequelae of intracerebral haemorrhage

Outcome	Code type	Code	Code Description
Stroke or TIA	ICD10	I69.2	Sequelae of other nontraumatic intracranial haemorrhage
Stroke or TIA	ICD10	I69.3	Sequelae of cerebral infarction
Stroke or TIA	ICD10	I69.4	Sequelae of stroke, not specified as haemorrhage or infarction
Stroke or TIA	ICD10	I69.8	Sequelae of other and unspecified cerebrovascular diseases
