# Supplementary material for: Antidepressant use and risk of cardiovascular outcomes in people aged 20 to 64: cohort study using primary care database
Source: BMJ. 2016 Mar 22;352:i1350. doi: 10.1136/bmj.i1350 (PMC4804126; doi:10.1136/bmj.i1350)
Supplement: Supplementary file 2 — Supplementary tables [file couc027737.ww2_default.pdf]

Antidepressant use and risk of cardiovascular outcomes in people aged 20 to 64: cohort study using primary care database

SUPPLEMENTARY TABLES

Table A	Numbers of prescriptions for different antidepressants by dose category
Table B	Adjusted hazard ratios for arrhythmia, myocardial infarction and stroke or transient ischaemic attack by antidepressant class, dose, and individual drug over 5 years follow-up with SSRIs as reference category for analysis of antidepressant class, mid-dose SSRIs as reference category for analysis of antidepressant dose and citalopram as reference category for analysis of individual drugs.
Table C	Unadjusted and adjusted hazard ratios for arrhythmia by citalopram split into 3 dose categories for 5 years follow-up.
Table D	Unadjusted and adjusted hazard ratios for arrhythmia by antidepressant class, dose and individual drug for 5 years follow-up, excluding untreated patients.
Table E	Unadjusted and adjusted hazard ratios for arrhythmia by antidepressant class, dose and individual drug for total follow-up.
Table F	Adjusted hazard ratios for arrhythmia, myocardial infarction and stroke or transient ischaemic attack by antidepressant class, with separate hazard ratios for 0-1, 1-3 and 3-5 years of follow-up
Table G	Adjusted hazard ratios for arrhythmia, myocardial infarction and stroke or transient ischaemic attack by individual antidepressant drug with separate hazard ratios for 0-1, 1-3 and 3-5 years of follow-up
Table H	Unadjusted and adjusted hazard ratios for myocardial infarction by antidepressant class, dose and individual drug for 5 years follow-up, excluding untreated patients.
Table I	Unadjusted and adjusted hazard ratios for myocardial infarction by antidepressant class, dose and individual drug for total follow-up.
Table J	Unadjusted and adjusted hazard ratios for stroke/ transient ischaemic attack by antidepressant class, dose and individual drug for 5 years follow-up, excluding untreated patients.
Table K	Unadjusted and adjusted hazard ratios for stroke/ transient ischaemic attack by antidepressant class, dose and individual drug for total follow-up.
Table L	Adjusted hazard ratios for arrhythmia by antidepressant class, and individual drug over 5 years adjusting for different subsets of confounders in blocks
Table M	Adjusted hazard ratios for myocardial infarction by antidepressant class, and individual drug over 5 years adjusting for different subsets of confounders in blocks
Table N	Adjusted hazard ratios for stroke/ transient ischaemic attack by antidepressant class, and individual drug over 5 years adjusting for different subsets of confounders in blocks

Table A Numbers of prescriptions for different antidepressant drugs by dose category

Antidepressant drug	n[1]	%[2]	DDD value (mg/day)	Actual dose prescribed (mg/day)[3] Median	IQR
Tricyclic and related antidepressants (TCA)					
Amitriptyline	236,416	7.3	75	25	15 to 50
Dosulepin	125,302	3.9	150	75	50 to 150
Lofepramine	47,414	1.5	105	140	140 to 210
Trazodone	30,912	1.0	300	125	75 to 150
Selective serotonin reuptake inhibitors (SSRI)					
Citalopram	1,023,255	31.5	20	20	20 to 20
Escitalopram	139,190	4.3	10	10	10 to 20
Fluoxetine	778,285	23.9	20	20	20 to 20
Paroxetine	159,389	4.9	20	20	20 to 30
Sertraline	213,749	6.6	50	50	50 to 100
Other antidepressants					
Mirtazapine	142,400	4.4	30	30	15 to 45
Venlafaxine	205,984	6.3	100	112.5	75 to 150
All other antidepressants	66,553	2.0	-		
Combined antidepressants[4]	83,784	2.6	-		
Total prescriptions	3,252,633				

[1] Number of prescriptions, where prescriptions for the same drug issued on the same day count as a single prescription and the doses have been summed.
[2] Percentage out of total number of prescriptions= 3,252,633
[3] 5.0% of prescriptions had missing information on dosage.
[4] Combined prescriptions for different antidepressant drugs are considered as a single prescription in this table.
DDD = defined daily dose value for the antidepressant drug

Table B Adjusted hazard ratios for arrhythmia, myocardial infarction and stroke or transient ischaemic attack by antidepressant class, dose, and individual drug over 5 years follow-up with SSRIs as reference category for analysis of antidepressant class, mid-dose SSRIs as reference category for analysis of antidepressant dose and citalopram as reference category for analysis of individual drugs

	Arrhythmia			Myocardial infarction			Stroke/TIA		
	Adjusted hazard ratio[1]	95% CI	P value	Adjusted hazard ratio[1]	95% CI	P value	Adjusted hazard ratio[1]	95% CI	P value
**Antidepressant class**									
SSRIs	1.00			1.00			1,00		
TCAs	1.29	(1.03 to 1.61)	0.03	1.42	(1.08 to 1.86)	0.012	1.14	(0.89 to 1.46)	0.28
Other antidepressants	1.44	(1.12 to 1.85)	0.005	1.18	(0.81 to 1.71)	0.39	1.11	(0.82 to 1.49)	0.51
Combined antidepressants	1.26	(0.64 to 2.51)	0.51	0.67	(0.21 to 2.11)	0.49	1.42	(0.78 to 2.59)	0.25
No current use	1.18	(1.03 to 1.37)	0.02	1.18	(1.00 to 1.41)	0.06	0.92	(0.79 to 1.07)	0.28
**Antidepressant class and dose categories**									
SSRIs:									
≤ 0.5 DDD	1.17	(0.80 to 1.73)	0.42	1.32	(0.76 to 2.29)	0.32	1.05	(0.68 to 1.63)	0.81
>0.5 DDD/≤ 1.0 DDD	1.00			1.00			1.00		
> 1.0 DDD	1.24	(0.93 to 1.64)	0.14	1.59	(1.13 to 2.22)	0.008	1.15	(0.87 to 1.51)	0.32
TCAs:									
≤ 0.5 DDD	1.13	(0.83 to 1.53)	0.46	1.40	(0.95 to 2.07)	0.09	1.04	(0.76 to 1.42)	0.83
>0.5 DDD/≤ 1.0 DDD	1.70	(1.13 to 2.54)	0.010	1.76	(1.05 to 2.94)	0.03	1.49	(1.00 to 2.24)	0.05
> 1.0 DDD	1.66	(0.97 to 2.86)	0.07	2.18	(1.15 to 4.14)	0.02	1.43	(0.78 to 2.62)	0.25
Others:									
≤ 0.5 DDD	1.23	(0.65 to 2.35)	0.52	2.46	(1.24 to 4.87)	0.010	1.45	(0.77 to 2.72)	0.25
>0.5 DDD/≤ 1.0 DDD	1.46	(1.01 to 2.12)	0.04	0.70	(0.34 to 1.43)	0.33	0.95	(0.61 to 1.50)	0.84
> 1.0 DDD	1.62	(1.04 to 2.52)	0.03	1.51	(0.83 to 2.76)	0.18	1.04	(0.60 to 1.78)	0.90
No current use	1.26	(1.06 to 1.49)	0.007	1.37	(1.10 to 1.69)	0.005	0.94	(0.80 to 1.11)	0.47
**Antidepressant drug**									
SSRIs:									
Citalopram	1.00			1.00			1.00		
Escitalopram	1.23	(0.80 to 1.89)	0.34	0.88	(0.46 to 1.69)	0.69	0.91	(0.55 to 1.50)	0.71
Fluoxetine	0.86	(0.67 to 1.11)	0.25	0.83	(0.58 to 1.17)	0.29	1.07	(0.84 to 1.35)	0.59
Paroxetine	1.13	(0.74 to 1.73)	0.56	0.86	(0.48 to 1.55)	0.62	0.90	(0.55 to 1.46)	0.66

Sertraline	1.13	(0.77 to 1.66)	0.53	1.45	(0.89 to 2.37)	0.14	1.18	(0.80 to 1.75)	0.41
TCAs:									
Amitriptyline	1.35	(0.99 to 1.84)	0.06	1.33	(0.88 to 2.00)	0.17	1.27	(0.91 to 1.77)	0.16
Dosulepin	1.08	(0.70 to 1.68)	0.73	1.33	(0.79 to 2.24)	0.28	1.09	(0.71 to 1.69)	0.69
Lofepramine	1.94	(1.16 to 3.27)	0.012	2.30	(1.24 to 4.25)	0.008	1.65	(0.88 to 3.09)	0.12
Trazodone	0.84	(0.30 to 2.30)	0.73	0.65	(0.16 to 2.73)	0.56	0.40	(0.10 to 1.57)	0.19
Others:									
Mirtazapine	1.40	(0.92 to 2.12)	0.11	1.49	(0.90 to 2.48)	0.12	1.27	(0.81 to 1.99)	0.29
Venlafaxine	1.48	(1.03 to 2.13)	0.03	1.01	(0.57 to 1.79)	0.98	0.99	(0.61 to 1.59)	0.95
All other antidepressants	0.85	(0.41 to 1.76)	0.67	0.59	(0.19 to 1.89)	0.38	0.94	(0.46 to 1.94)	0.88
Combined antidepressants	1.24	(0.62 to 2.48)	0.54	0.64	(0.20 to 2.06)	0.46	1.45	(0.79 to 2.66)	0.23
No current use	1.17	(0.97 to 1.40)	0.11	1.14	(0.90 to 1.45)	0.29	0.94	(0.77 to 1.15)	0.55

SSRIs=selective serotonin reuptake inhibitors; TCAs=tricyclic and related antidepressants.

[1] Adjusted for age, sex, year of diagnosis of depression, severity of depression, deprivation, smoking status, alcohol intake, ethnic group (white/not recorded or non-white), coronary heart disease, diabetes, hypertension, cancer, epilepsy/seizures, hypothyroidism, osteoarthritis, asthma/chronic obstructive airways disease, stroke/TIA (except for the stroke/TIA outcome), rheumatoid arthritis, osteoporosis, liver disease, renal disease, obsessive-compulsive disorder, statins, NSAIDS, aspirin, antihypertensive drugs, anticonvulsants, hypnotics/anxiolytics, oral contraceptives, hormone replacement therapy, antipsychotics, bisphosphonates, anticoagulants.

Table C Unadjusted and adjusted hazard ratios for arrhythmia over 5 years follow-up with citalopram dose categorised into three categories

			Unadjusted			Adjusted analysis[1]		
Daily dose of citalopram	No of events*	Person years*	HR	95% CI	P	HR	95% CI	P
No current use	887	568,365	1.00			1.00		
Citalopram: ≤20 mg/day	115	72,340	1.05	(0.86 to 1.28)	0.65	0.82	(0.67 to 1.01)	0.07
Citalopram: 20-39 mg/day	6	3,947	0.99	(0.45 to 2.19)	0.99	0.93	(0.42 to 2.06)	0.87
Citalopram: ≥40 mg/day	28	13,907	1.35	(0.89 to 2.05)	0.16	1.11	(0.72 to 1.71)	0.62

* Based on numbers in adjusted analysis

[1] Adjusted for age, sex, year of diagnosis of depression, severity of depression, deprivation, smoking status, alcohol intake, ethnic group (white/not recorded; non-white), coronary heart disease, diabetes, hypertension, cancer, epilepsy/seizures, hypothyroidism, osteoarthritis, asthma/chronic obstructive airways disease, stroke/TIA, rheumatoid arthritis, osteoporosis, liver disease, renal disease, obsessive-compulsive disorder, statins, NSAIDS, aspirin, antihypertensive drugs, anticonvulsants, hypnotics/anxiolytics, oral contraceptives, hormone replacement therapy, antipsychotics, bisphosphonates, anticoagulants.

Table D Unadjusted and adjusted hazard ratios for arrhythmia by antidepressant class, dose and individual drug for 5 years follow-up, excluding untreated patients

		Unadjusted analysis			Adjusted analysis[1]		
	No of events*	HR	95% CI	P	HR	95% CI	P
**Antidepressant class**							
No current use	731	1.00			1.00		
TCAs	102	1.59	(1.28 to 1.97)	<0.001	1.08	(0.86 to 1.34)	0.52
SSRIs	352	1.02	(0.88 to 1.18)	0.80	0.83	(0.71 to 0.96)	0.012
Other antidepressants	68	1.55	(1.23 to 1.96)	<0.001	1.21	(0.95 to 1.53)	0.12
Combined antidepressants	10	1.49	(0.76 to 2.91)	0.25	1.07	(0.55 to 2.08)	0.85
**Antidepressant class and dose categories**							
No current use	731	1.00			1.00		
TCAs:							
≤ 0.5 DDD	51	1.36	(1.02 to 1.82)	0.04	0.88	(0.66 to 1.18)	0.39
>0.5 DDD/≤ 1.0 DDD	26	2.03	(1.38 to 2.97)	<0.001	1.34	(0.90 to 1.99)	0.15
> 1.0 DDD	14	1.66	(0.98 to 2.81)	0.06	1.31	(0.76 to 2.25)	0.33
SSRIs:							
≤ 0.5 DDD	30	1.18	(0.81 to 1.71)	0.38	0.91	(0.62 to 1.32)	0.62
>0.5 DDD/≤ 1.0 DDD	236	0.96	(0.80 to 1.14)	0.63	0.77	(0.65 to 0.92)	0.004
> 1.0 DDD	75	1.17	(0.91 to 1.5)	0.22	0.97	(0.75 to 1.25)	0.80
Others:							
≤ 0.5 DDD	9	1.40	(0.74 to 2.64)	0.31	0.96	(0.51 to 1.82)	0.90
>0.5 DDD/≤ 1.0 DDD	31	1.52	(1.08 to 2.15)	0.02	1.15	(0.81 to 1.64)	0.44
> 1.0 DDD	20	1.50	(0.98 to 2.31)	0.06	1.28	(0.84 to 1.97)	0.25
**Antidepressant drug**							
No current use	731	1.00			1.00		
TCAs:							
Amitriptyline	54	1.75	(1.32 to 2.33)	<0.001	1.15	(0.86 to 1.53)	0.35
Dosulepin	25	1.31	(0.86 to 1.98)	0.21	0.92	(0.60 to 1.39)	0.68
Lofepramine	16	2.09	(1.27 to 3.45)	0.004	1.65	(0.99 to 2.74)	0.053
Trazodone	4	1.33	(0.55 to 3.21)	0.53	0.71	(0.26 to 1.93)	0.50
SSRIs:							
Citalopram	153	1.07	(0.89 to 1.29)	0.47	0.84	(0.69 to 1.01)	0.07
Escitalopram	26	1.33	(0.91 to 1.94)	0.15	1.04	(0.69 to 1.55)	0.86
Fluoxetine	111	0.86	(0.68 to 1.08)	0.20	0.72	(0.57 to 0.91)	0.005
Paroxetine	29	1.12	(0.76 to 1.65)	0.56	0.96	(0.65 to 1.42)	0.84
Sertraline	33	1.15	(0.80 to 1.66)	0.45	0.95	(0.65 to 1.38)	0.79
Others:							
Mirtazapine	27	1.74	(1.19 to 2.53)	0.004	1.19	(0.81 to 1.76)	0.37
Venlafaxine	36	1.46	(1.04 to 2.05)	0.03	1.27	(0.90 to 1.78)	0.18
All other antidepressants	8	1.02	(0.51 to 2.03)	0.96	0.72	(0.36 to 1.44)	0.36
Combined antidepressants	10	1.49	(0.76 to 2.90)	0.25	1.06	(0.54 to 2.08)	0.85

* Based on numbers in adjusted analysis

DDD= defined daily dose

[1] Adjusted for age, sex, year of diagnosis of depression, severity of depression, deprivation, smoking status, alcohol intake, ethnic group (white/not recorded or non-white), coronary heart disease, diabetes, hypertension, cancer, epilepsy/seizures, hypothyroidism, osteoarthritis, asthma/chronic obstructive airways disease, stroke/TIA, rheumatoid arthritis, osteoporosis, liver disease, renal disease, obsessive-compulsive disorder, statins, NSAIDS, aspirin, antihypertensive drugs, anticonvulsants, hypnotics/anxiolytics, oral contraceptives, hormone replacement therapy, antipsychotics, bisphosphonates, anticoagulants.

Table E Unadjusted and adjusted hazard ratios for arrhythmia by antidepressant class, dose and individual drug for total follow-up period

		Unadjusted analysis			Adjusted analysis[1]		
	No of events*	HR	95% CI	P	HR	95% CI	P
**Antidepressant class**							
No current use	1517	1.00			1.00		
TCAs	165	1.60	(1.37 to 1.88)	<0.001	1.10	(0.93 to 1.30)	0.25
SSRIs	462	0.93	(0.83 to 1.05)	0.24	0.79	(0.71 to 0.89)	<0.001
Other antidepressants	104	1.48	(1.23 to 1.79)	<0.001	1.21	(1.00 to 1.46)	0.052
Combined antidepressants	17	1.26	(0.77 to 2.05)	0.35	0.94	(0.58 to 1.54)	0.82
**Antidepressant class and dose categories**							
No current use	1517	1.00			1.00		
TCAs:							
≤ 0.5 DDD	85	1.40	(1.12 to 1.73)	0.003	0.92	(0.74 to 1.15)	0.48
>0.5 DDD/≤ 1.0 DDD	42	2.03	(1.51 to 2.73)	<0.001	1.37	(1.01 to 1.85)	0.045
> 1.0 DDD	22	1.79	(1.18 to 2.71)	0.006	1.43	(0.93 to 2.20)	0.11
SSRIs:							
≤ 0.5 DDD	41	1.11	(0.82 to 1.50)	0.52	0.88	(0.65 to 1.19)	0.41
>0.5 DDD/≤ 1.0 DDD	297	0.88	(0.77 to 1.01)	0.07	0.74	(0.64 to 0.85)	<0.001
> 1.0 DDD	107	1.02	(0.83 to 1.25)	0.87	0.90	(0.73 to 1.11)	0.33
Others:							
≤ 0.5 DDD	11	1.03	(0.58 to 1.82)	0.92	0.77	(0.44 to 1.37)	0.38
>0.5 DDD/≤ 1.0 DDD	47	1.50	(1.12 to 2.00)	0.006	1.18	(0.87 to 1.59)	0.28
> 1.0 DDD	36	1.57	(1.14 to 2.18)	0.006	1.40	(1.02 to 1.93)	0.037
**Antidepressant drug**							
No current use	1517	1.00			1.00		
TCAs:							
Amitriptyline	92	1.67	(1.35 to 2.05)	<0.001	1.14	(0.92 to 1.41)	0.24
Dosulepin	33	1.28	(0.90 to 1.82)	0.17	0.89	(0.63 to 1.27)	0.53
Lofepramine	24	2.42	(1.62 to 3.61)	<0.001	1.95	(1.31 to 2.88)	0.001
Trazodone	10	1.74	(0.95 to 3.18)	0.07	1.11	(0.59 to 2.08)	0.74
SSRIs:							
Citalopram	204	0.96	(0.82 to 1.13)	0.65	0.80	(0.68 to 0.94)	0.008
Escitalopram	32	1.13	(0.79 to 1.62)	0.49	0.92	(0.63 to 1.32)	0.64
Fluoxetine	143	0.82	(0.68 to 0.98)	0.03	0.72	(0.60 to 0.86)	<0.001
Paroxetine	36	1.02	(0.72 to 1.45)	0.90	0.87	(0.61 to 1.24)	0.44
Sertraline	47	1.05	(0.78 to 1.41)	0.74	0.91	(0.67 to 1.23)	0.54
Others:							
Mirtazapine	42	1.61	(1.20 to 2.15)	0.001	1.20	(0.89 to 1.62)	0.23
Venlafaxine	53	1.40	(1.07 to 1.83)	0.013	1.24	(0.95 to 1.62)	0.12
All other antidepressants	15	1.14	(0.69 to 1.87)	0.60	0.74	(0.43 to 1.26)	0.27
Combined antidepressants	17	1.26	(0.77 to 2.05)	0.35	0.94	(0.58 to 1.54)	0.81

* Based on numbers in adjusted analysis

DDD= defined daily dose

[1] Adjusted for age, sex, year of diagnosis of depression, severity of depression, deprivation, smoking status, alcohol intake, ethnic group (white/not recorded or non-white), coronary heart disease, diabetes, hypertension, cancer, epilepsy/seizures, hypothyroidism, osteoarthritis, asthma/chronic obstructive airways disease, stroke/TIA, rheumatoid arthritis, osteoporosis, liver disease, renal disease, obsessive-compulsive disorder, statins, NSAIDS, aspirin, antihypertensive drugs, anticonvulsants, hypnotics/anxiolytics, oral contraceptives, hormone replacement therapy, antipsychotics, bisphosphonates, anticoagulants.

Table F Adjusted hazard ratios for arrhythmia, myocardial infarction and stroke or transient ischaemic attack by antidepressant class split over years 0-1, 1-3, and 3-5 years of follow-up

	**Arrhythmia**			**Myocardial infarction**			**Stroke/TIA**		
Drug class by year of follow-up	Adjusted hazard ratio[1]	95% CI	P	Adjusted hazard ratio[1]	95% CI	P	Adjusted hazard ratio[1]	95% CI	P
TCAs:									
0-1 years	1.16	(0.81 to 1.66)	0.42	1.07	(0.71 to 1.62)	0.73	1.03	(0.70 to 1.51)	0.90
1-3 years	1.23	(0.89 to 1.70)	0.21	1.24	(0.82 to 1.88)	0.30	1.08	(0.72 to 1.62)	0.70
3-5 years	0.85	(0.56 to 1.27)	0.42	1.12	(0.68 to 1.85)	0.64	1.62	(1.10 to 2.37)	0.014
SSRIs:									
0-1 years	0.85	(0.66 to 1.10)	0.21	0.59	(0.43 to 0.81)	0.001	0.84	(0.64 to 1.10)	0.21
1-3 years	0.90	(0.73 to 1.10)	0.31	0.88	(0.68 to 1.15)	0.36	1.09	(0.87 to 1.37)	0.45
3-5 years	0.79	(0.62 to 1.00)	0.052	1.14	(0.83 to 1.57)	0.41	1.37	(1.06 to 1.75)	0.014
Others:									
0-1 years	1.36	(0.86 to 2.15)	0.19	0.82	(0.43 to 1.58)	0.55	1.18	(0.71 to 1.95)	0.53
1-3 years	1.09	(0.73 to 1.61)	0.68	0.97	(0.56 to 1.69)	0.91	1.37	(0.90 to 2.08)	0.15
3-5 years	1.28	(0.83 to 1.98)	0.27	1.14	(0.62 to 2.10)	0.68	0.90	(0.50 to 1.62)	0.72

SSRIs=selective serotonin reuptake inhibitors; TCAs=tricyclic and related antidepressants.

[1] Adjusted for age, sex, year of diagnosis of depression, severity of depression, deprivation, smoking status, alcohol intake, ethnic group (white/not recorded or non-white), coronary heart disease, diabetes, hypertension, cancer, epilepsy/seizures, hypothyroidism, osteoarthritis, asthma/chronic obstructive airways disease, stroke/TIA (except for the stroke/TIA outcome), rheumatoid arthritis, osteoporosis, liver disease, renal disease, obsessive-compulsive disorder, statins, NSAIDS, aspirin, antihypertensive drugs, anticonvulsants, hypnotics/anxiolytics, oral contraceptives, hormone replacement therapy, antipsychotics, bisphosphonates, anticoagulants. Model also included terms for combined antidepressant treatment.

Reference group is no current use of antidepressants in each specific time interval.

Table G Adjusted hazard ratios for arrhythmia, myocardial infarction and stroke or transient ischaemic attack for five antidepressant drugs split over years 0-1, 1-3, and 3-5 years of follow-up

	**Arrhythmia**			**Myocardial infarction**			**Stroke/TIA**		
Drug by year of follow-up	Adjusted hazard ratio[1]	95% CI	P	Adjusted hazard ratio[1]	95% CI	P	Adjusted hazard ratio[1]	95% CI	P
Amitriptyline									
0-1 years	1.19	(0.71 to 2.00)	0.50	0.77	(0.37 to 1.56)	0.46	1.02	(0.59 to 1.74)	0.95
1-3 years	1.31	(0.85 to 2.02)	0.22	1.90	(1.18 to 3.06)	0.009	1.29	(0.78 to 2.13)	0.31
3-5 years	0.98	(0.59 to 1.62)	0.94	0.68	(0.29 to 1.60)	0.38	1.70	(1.03 to 2.80)	0.037
Citalopram									
0-1 years	0.81	(0.58 to 1.12)	0.20	0.62	(0.40 to 0.96)	0.030	0.75	(0.52 to 1.07)	0.12
1-3 years	1.07	(0.83 to 1.39)	0.60	0.78	(0.52 to 1.18)	0.24	1.00	(0.73 to 1.37)	0.98
3-5 years	0.66	(0.46 to 0.97)	0.032	1.37	(0.91 to 2.04)	0.13	1.57	(1.14 to 2.17)	0.006
Fluoxetine									
0-1 years	0.77	(0.54 to 1.09)	0.14	0.44	(0.27 to 0.73)	0.001	1.06	(0.75 to 1.48)	0.75
1-3 years	0.70	(0.50 to 0.99)	0.046	0.98	(0.66 to 1.48)	0.94	1.09	(0.79 to 1.50)	0.61
3-5 years	0.78	(0.53 to 1.16)	0.22	0.79	(0.43 to 1.46)	0.46	1.08	(0.69 to 1.69)	0.75
Sertraline									
0-1 years	1.23	(0.72 to 2.08)	0.45	1.22	(0.66 to 2.25)	0.54	0.65	(0.31 to 1.39)	0.27
1-3 years	0.80	(0.43 to 1.49)	0.49	0.90	(0.41 to 1.98)	0.80	1.60	(0.94 to 2.73)	0.08
3-5 years	0.89	(0.44 to 1.80)	0.74	1.70	(0.79 to 3.65)	0.17	1.61	(0.84 to 3.11)	0.15
Venlafaxine									
0-1 years	1.61	(0.87 to 2.98)	0.13	0.84	(0.32 to 2.23)	0.73	0.50	(0.16 to 1.55)	0.23
1-3 years	1.06	(0.63 to 1.81)	0.82	0.59	(0.22 to 1.61)	0.31	1.29	(0.72 to 2.33)	0.39
3-5 years	1.32	(0.76 to 2.31)	0.32	1.30	(0.57 to 2.93)	0.53	1.18	(0.57 to 2.47)	0.66
All others (including combined)									
0-1 years	1.16	(0.85 to 1.57)	0.35	0.92	(0.62 to 1.35)	0.66	0.97	(0.68 to 1.38)	0.86
1-3 years	1.00	(0.75 to 1.34)	0.98	0.89	(0.59 to 1.32)	0.56	1.18	(0.84 to 1.64)	0.34
3-5 years	0.98	(0.69 to 1.39)	0.90	1.08	(0.68 to 1.72)	0.75	1.17	(0.79 to 1.74)	0.42

[1] Adjusted for age, sex, year of diagnosis of depression, severity of depression, deprivation, smoking status, alcohol intake, ethnic group (white/not recorded or non-white), coronary heart disease, diabetes, hypertension, cancer, epilepsy/seizures, hypothyroidism, osteoarthritis, asthma/chronic obstructive airways disease, stroke/TIA (except for the stroke/TIA outcome), rheumatoid arthritis, osteoporosis, liver disease, renal disease, obsessive-compulsive disorder, statins, NSAIDS, aspirin, antihypertensive drugs, anticonvulsants, hypnotics/anxiolytics, oral contraceptives, hormone replacement therapy, antipsychotics, bisphosphonates, anticoagulants.
Reference group is no current use of antidepressants in each specific time interval.

Table H Unadjusted and adjusted hazard ratios for myocardial infarction by antidepressant class, dose and individual drug for 5 years follow-up, excluding untreated patients

		Unadjusted analysis			Adjusted analysis[1]		
	No of events*	HR	95% CI	P	HR	95% CI	P
**Antidepressant class**							
No current use	397	1.00			1.00		
TCAs	63	1.83	(1.43 to 2.35)	<0.001	1.19	(0.93 to 1.52)	0.16
SSRIs	182	1.02	(0.86 to 1.23)	0.79	0.84	(0.70 to 1.00)	0.051
Other antidepressants	33	1.38	(0.97 to 1.98)	0.08	0.98	(0.68 to 1.40)	0.89
Combined antidepressants	3	0.83	(0.27 to 2.55)	0.75	0.56	(0.18 to 1.72)	0.31
**Antidepressant class and dose categories**							
No current use	397	1.00			1.00		
TCAs:							
≤ 0.5 DDD	31	1.60	(1.12 to 2.28)	0.010	1.02	(0.72 to 1.46)	0.89
>0.5 DDD/≤ 1.0 DDD	15	2.14	(1.30 to 3.52)	0.003	1.28	(0.77 to 2.11)	0.34
> 1.0 DDD	10	2.24	(1.2 to 4.17)	0.011	1.57	(0.84 to 2.94)	0.15
SSRIs:							
≤ 0.5 DDD	14	1.13	(0.68 to 1.86)	0.64	0.96	(0.57 to 1.61)	0.87
>0.5 DDD/≤ 1.0 DDD	110	0.90	(0.72 to 1.12)	0.34	0.73	(0.58 to 0.91)	0.005
> 1.0 DDD	50	1.45	(1.09 to 1.91)	0.010	1.14	(0.85 to 1.52)	0.39
Others:							
≤ 0.5 DDD	9	2.64	(1.37 to 5.10)	0.004	1.77	(0.91 to 3.43)	0.09
>0.5 DDD/≤ 1.0 DDD	8	0.71	(0.36 to 1.42)	0.34	0.50	(0.25 to 1.01)	0.052
> 1.0 DDD	11	1.52	(0.84 to 2.76)	0.17	1.08	(0.59 to 1.96)	0.81
**Antidepressant drug**							
No current use	397				1.00		
TCAs:							
Amitriptyline	30	1.84	(1.28 to 2.63)	0.001	1.16	(0.81 to 1.66)	0.41
Dosulepin	18	1.80	(1.14 to 2.83)	0.011	1.16	(0.74 to 1.83)	0.51
Lofepramine	11	2.74	(1.54 to 4.86)	0.001	2.00	(1.13 to 3.56)	0.018
Trazodone	2	1.01	(0.25 to 4.05)	0.99	0.57	(0.14 to 2.29)	0.43
SSRIs:							
Citalopram	79	1.05	(0.82 to 1.34)	0.69	0.87	(0.68 to 1.12)	0.27
Escitalopram	10	0.89	(0.48 to 1.67)	0.72	0.76	(0.40 to 1.42)	0.38
Fluoxetine	56	0.89	(0.67 to 1.19)	0.43	0.72	(0.53 to 0.97)	0.031
Paroxetine	13	1.02	(0.60 to 1.73)	0.94	0.75	(0.43 to 1.29)	0.29
Sertraline	23	1.52	(1.00 to 2.31)	0.048	1.25	(0.82 to 1.91)	0.31
Others:							
Mirtazapine	19	2.20	(1.36 to 3.54)	0.001	1.29	(0.79 to 2.10)	0.30
Venlafaxine	14	1.05	(0.62 to 1.79)	0.85	0.86	(0.51 to 1.47)	0.59
All other antidepressants	3	0.72	(0.24 to 2.20)	0.57	0.51	(0.17 to 1.56)	0.24
Combined antidepressants	3	0.83	(0.27 to 2.55)	0.75	0.56	(0.18 to 1.72)	0.31

* Based on numbers in adjusted analysis

DDD= defined daily dose

[1] Adjusted for age, sex, year of diagnosis of depression, severity of depression, deprivation, smoking status, alcohol intake, ethnic group (white/not recorded or non-white), coronary heart disease, diabetes, hypertension, cancer, epilepsy/seizures, hypothyroidism, osteoarthritis, asthma/chronic obstructive airways disease, stroke/TIA, rheumatoid arthritis, osteoporosis, liver disease, renal disease, obsessive-compulsive disorder, statins, NSAIDS, aspirin, antihypertensive drugs, anticonvulsants, hypnotics/anxiolytics, oral contraceptives, hormone replacement therapy, antipsychotics, bisphosphonates, anticoagulants.

Table I Unadjusted and adjusted hazard ratios for myocardial infarction by antidepressant class, dose and individual drug for total follow-up period

		Unadjusted analysis			Adjusted analysis[1]		
	No of events*	HR	95% CI	P	HR	95% CI	P
**Antidepressant class**							
No current use	783	1.00			1.00		
TCAs	93	1.78	(1.44 to 2.19)	<0.001	1.18	(0.96 to 1.45)	0.12
SSRIs	260	1.02	(0.89 to 1.18)	0.75	0.88	(0.76 to 1.02)	0.080
Other antidepressants	60	1.66	(1.28 to 2.17)	<0.001	1.22	(0.93 to 1.60)	0.16
Combined antidepressants	8	1.17	(0.60 to 2.30)	0.65	0.81	(0.41 to 1.60)	0.55
**Antidepressant class and dose categories**							
No current use	783	1.00			1.00		
TCAs:							
≤ 0.5 DDD	46	1.51	(1.11 to 2.04)	0.008	0.98	(0.73 to 1.33)	0.90
>0.5 DDD/≤ 1.0 DDD	25	2.38	(1.61 to 3.53)	<0.001	1.44	(0.97 to 2.15)	0.072
> 1.0 DDD	14	2.23	(1.32 to 3.75)	0.003	1.62	(0.97 to 2.71)	0.065
SSRIs:							
≤ 0.5 DDD	21	1.11	(0.73 to 1.69)	0.63	0.97	(0.63 to 1.48)	0.87
>0.5 DDD/≤ 1.0 DDD	160	0.94	(0.79 to 1.12)	0.52	0.80	(0.67 to 0.96)	0.015
> 1.0 DDD	71	1.31	(1.03 to 1.67)	0.030	1.11	(0.87 to 1.43)	0.41
Others:							
≤ 0.5 DDD	13	2.39	(1.39 to 4.11)	0.002	1.76	(1.02 to 3.04)	0.041
>0.5 DDD/≤ 1.0 DDD	16	1.00	(0.62 to 1.61)	1.00	0.71	(0.44 to 1.15)	0.17
> 1.0 DDD	25	2.13	(1.40 to 3.23)	<0.001	1.56	(1.02 to 2.40)	0.040
**Antidepressant drug**							
No current use	783	1.00			1.00		
TCAs:							
Amitriptyline	49	1.75	(1.33 to 2.31)	<0.001	1.14	(0.86 to 1.51)	0.36
Dosulepin	25	1.90	(1.29 to 2.79)	0.001	1.27	(0.86 to 1.87)	0.22
Lofepramine	13	2.55	(1.51 to 4.30)	<0.001	1.90	(1.15 to 3.12)	0.012
Trazodone	2	0.68	(0.17 to 2.70)	0.58	0.38	(0.09 to 1.52)	0.17
SSRIs:							
Citalopram	113	1.04	(0.85 to 1.27)	0.70	0.91	(0.74 to 1.12)	0.36
Escitalopram	13	0.89	(0.52 to 1.53)	0.67	0.79	(0.46 to 1.35)	0.38
Fluoxetine	78	0.87	(0.69 to 1.10)	0.25	0.74	(0.58 to 0.95)	0.019
Paroxetine	21	1.16	(0.75 to 1.78)	0.51	0.87	(0.56 to 1.35)	0.53
Sertraline	33	1.44	(1.02 to 2.04)	0.039	1.27	(0.90 to 1.81)	0.18
Others:							
Mirtazapine	32	2.38	(1.67 to 3.40)	<0.001	1.48	(1.03 to 2.14)	0.036
Venlafaxine	27	1.39	(0.95 to 2.04)	0.093	1.15	(0.78 to 1.70)	0.48
All other antidepressants	7	1.04	(0.46 to 2.38)	0.92	0.80	(0.35 to 1.84)	0.60
Combined antidepressants	8	1.17	(0.60 to 2.30)	0.65	0.81	(0.41 to 1.60)	0.55

* Based on numbers in adjusted analysis

DDD= defined daily dose

[1] Adjusted for age, sex, year of diagnosis of depression, severity of depression, deprivation, smoking status, alcohol intake, ethnic group (white/not recorded or non-white), coronary heart disease, diabetes, hypertension, cancer, epilepsy/seizures, hypothyroidism, osteoarthritis, asthma/chronic obstructive airways disease, stroke/TIA, rheumatoid arthritis, osteoporosis, liver disease, renal disease, obsessive-compulsive disorder, statins, NSAIDS, aspirin, antihypertensive drugs, anticonvulsants, hypnotics/anxiolytics, oral contraceptives, hormone replacement therapy, antipsychotics, bisphosphonates, anticoagulants.

Table J Unadjusted and adjusted hazard ratios for stroke/TIA by antidepressant class, dose and individual drug for 5 years follow-up, excluding untreated patients

		Unadjusted analysis			Adjusted analysis[1]		
	No of events*	HR	95% CI	P	HR	95% CI	P
**Antidepressant class**							
No current use	525	1.00			1.00		
TCAs	90	1.93	(1.52 to 2.46)	<0.001	1.19	(0.93 to 1.51)	0.17
SSRIs	313	1.26	(1.08 to 1.48)	0.003	1.04	(0.89 to 1.21)	0.65
Other antidepressants	50	1.66	(1.25 to 2.20)	<0.001	1.15	(0.86 to 1.53)	0.36
Combined antidepressants	11	2.51	(1.42 to 4.43)	0.001	1.47	(0.81 to 2.65)	0.20
**Antidepressant class and dose categories**							
No current use	525	1.00			1.00		
TCAs:							
≤ 0.5 DDD	48	1.80	(1.32 to 2.45)	<0.001	1.05	(0.77 to 1.43)	0.75
>0.5 DDD/≤ 1.0 DDD	24	2.54	(1.72 to 3.77)	<0.001	1.51	(1.02 to 2.26)	0.042
> 1.0 DDD	12	2.00	(1.10 to 3.66)	0.024	1.44	(0.79 to 2.62)	0.24
SSRIs:							
≤ 0.5 DDD	24	1.33	(0.86 to 2.06)	0.20	1.07	(0.69 to 1.66)	0.76
>0.5 DDD/≤ 1.0 DDD	216	1.25	(1.05 to 1.49)	0.011	1.01	(0.85 to 1.20)	0.88
> 1.0 DDD	66	1.40	(1.08 to 1.81)	0.012	1.16	(0.89 to 1.51)	0.28
Others:							
≤ 0.5 DDD	10	2.18	(1.17 to 4.07)	0.014	1.47	(0.78 to 2.76)	0.23
>0.5 DDD/≤ 1.0 DDD	20	1.46	(0.96 to 2.24)	0.08	0.97	(0.62 to 1.51)	0.88
> 1.0 DDD	13	1.36	(0.80 to 2.31)	0.26	1.05	(0.61 to 1.79)	0.87
**Antidepressant drug**							
No current use	525	1.00			1.00		
TCAs:							
Amitriptyline	49	2.21	(1.64 to 2.99)	<0.001	1.29	(0.95 to 1.75)	0.10
Dosulepin	24	1.76	(1.17 to 2.66)	0.007	1.11	(0.74 to 1.68)	0.62
Lofepramine	12	2.21	(1.21 to 4.05)	0.010	1.66	(0.91 to 3.04)	0.10
Trazodone	2	0.75	(0.19 to 2.97)	0.68	0.41	(0.11 to 1.58)	0.20
SSRIs:							
Citalopram	128	1.21	(0.99 to 1.48)	0.06	1.01	(0.83 to 1.24)	0.89
Escitalopram	17	1.12	(0.68 to 1.84)	0.65	0.92	(0.56 to 1.52)	0.75
Fluoxetine	117	1.32	(1.07 to 1.62)	0.009	1.08	(0.88 to 1.33)	0.47
Paroxetine	21	1.18	(0.76 to 1.84)	0.46	0.91	(0.58 to 1.43)	0.68
Sertraline	30	1.50	(1.03 to 2.18)	0.032	1.20	(0.82 to 1.74)	0.35
Others:							
Mirtazapine	24	2.16	(1.46 to 3.20)	<0.001	1.29	(0.86 to 1.94)	0.22
Venlafaxine	21	1.30	(0.85 to 1.98)	0.23	1.00	(0.64 to 1.56)	1.00
All other antidepressants	8	1.43	(0.71 to 2.88)	0.32	0.96	(0.48 to 1.93)	0.90
Combined antidepressants	11	2.51	(1.42 to 4.43)	0.001	1.47	(0.81 to 2.65)	0.20

* Based on numbers in adjusted analysis

DDD= defined daily dose

[1] Adjusted for age, sex, year of diagnosis of depression, severity of depression, deprivation, smoking status, alcohol intake, ethnic group (white/not recorded or non-white), coronary heart disease, diabetes, hypertension, cancer, epilepsy/seizures, hypothyroidism, osteoarthritis, asthma/chronic obstructive airways disease, rheumatoid arthritis, osteoporosis, liver disease, renal disease, obsessive-compulsive disorder, statins, NSAIDS, aspirin, antihypertensive drugs, anticonvulsants, hypnotics/anxiolytics, oral contraceptives, hormone replacement therapy, antipsychotics, bisphosphonates, anticoagulants.

Table K Unadjusted and adjusted hazard ratios for stroke/TIA by antidepressant class, dose and individual drug for total follow-up period

		Unadjusted analysis			Adjusted analysis[1]		
	No of events*	HR	95% CI	P	HR	95% CI	P
**Antidepressant class**							
No current use	1082	1.00			1.00		
TCAs	138	1.92	(1.59 to 2.31)	<0.001	1.23	(1.01 to 1.49)	0.036
SSRIs	447	1.28	(1.14 to 1.44)	<0.001	1.10	(0.98 to 1.24)	0.12
Other antidepressants	81	1.63	(1.31 to 2.04)	<0.001	1.20	(0.96 to 1.50)	0.11
Combined antidepressants	21	2.22	(1.47 to 3.35)	<0.001	1.44	(0.94 to 2.20)	0.09
**Antidepressant class and dose categories**							
No current use	1082	1.00			1.00		
TCAs:							
≤ 0.5 DDD	80	1.89	(1.49 to 2.38)	<0.001	1.17	(0.92 to 1.48)	0.20
>0.5 DDD/≤ 1.0 DDD	31	2.14	(1.53 to 3.01)	<0.001	1.32	(0.93 to 1.86)	0.12
> 1.0 DDD	16	1.86	(1.12 to 3.09)	0.017	1.33	(0.78 to 2.26)	0.29
SSRIs:							
≤ 0.5 DDD	32	1.22	(0.83 to 1.80)	0.30	1.02	(0.69 to 1.49)	0.94
>0.5 DDD/≤ 1.0 DDD	293	1.25	(1.08 to 1.44)	0.002	1.05	(0.92 to 1.21)	0.46
> 1.0 DDD	112	1.50	(1.24 to 1.82)	<0.001	1.32	(1.08 to 1.61)	0.006
Others:							
≤ 0.5 DDD	14	1.86	(1.11 to 3.12)	0.018	1.37	(0.81 to 2.29)	0.24
>0.5 DDD/≤ 1.0 DDD	32	1.45	(1.03 to 2.05)	0.033	1.02	(0.72 to 1.46)	0.90
> 1.0 DDD	26	1.60	(1.07 to 2.42)	0.024	1.27	(0.85 to 1.90)	0.25
**Antidepressant drug**							
No current use	1082	1.00			1.00		
TCAs:							
Amitriptyline	79	2.05	(1.63 to 2.58)	<0.001	1.28	(1.01 to 1.62)	0.039
Dosulepin	31	1.72	(1.19 to 2.47)	0.004	1.10	(0.76 to 1.59)	0.62
Lofepramine	16	2.32	(1.40 to 3.86)	0.001	1.82	(1.09 to 3.04)	0.022
Trazodone	6	1.48	(0.66 to 3.29)	0.34	0.83	(0.37 to 1.83)	0.64
SSRIs:							
Citalopram	189	1.27	(1.08 to 1.49)	0.004	1.11	(0.95 to 1.30)	0.20
Escitalopram	28	1.40	(0.95 to 2.06)	0.089	1.16	(0.78 to 1.72)	0.47
Fluoxetine	154	1.25	(1.05 to 1.48)	0.011	1.08	(0.91 to 1.29)	0.38
Paroxetine	31	1.25	(0.88 to 1.79)	0.22	0.99	(0.68 to 1.42)	0.94
Sertraline	44	1.40	(1.03 to 1.90)	0.034	1.17	(0.85 to 1.61)	0.33
Others:							
Mirtazapine	35	1.90	(1.36 to 2.64)	<0.001	1.25	(0.90 to 1.75)	0.19
Venlafaxine	39	1.46	(1.05 to 2.01)	0.022	1.17	(0.84 to 1.63)	0.36
All other antidepressants	14	1.51	(0.90 to 2.55)	0.12	1.08	(0.65 to 1.81)	0.77
Combined antidepressants	21	2.22	(1.47 to 3.35)	<0.001	1.44	(0.94 to 2.20)	0.092

* Based on numbers in adjusted analysis

DDD= defined daily dose

[1] Adjusted for age, sex, year of diagnosis of depression, severity of depression, deprivation, smoking status, alcohol intake, ethnic group (white/not recorded or non-white), coronary heart disease, diabetes, hypertension, cancer, epilepsy/seizures, hypothyroidism, osteoarthritis, asthma/chronic obstructive airways disease, rheumatoid arthritis, osteoporosis, liver disease, renal disease, obsessive-compulsive disorder, statins, NSAIDS, aspirin, antihypertensive drugs, anticonvulsants, hypnotics/anxiolytics, oral contraceptives, hormone replacement therapy, antipsychotics, bisphosphonates, anticoagulants.

Table L Adjusted hazard ratios for arrhythmia by antidepressant class, and individual drug over 5 years follow-up adjusted for different subsets of confounding variables

	Unadjusted		Model A[1]		Model B[2]		Model C[3]		Full model[4]	
	Hazard ratio	95% CI	Adjusted hazard ratio	95% CI	Adjusted hazard ratio	95% CI	Adjusted hazard ratio	95% CI	Adjusted hazard ratio	95% CI
**Antidepressant class**										
No current use	1.00		1.00		1.00		1.00		1.00	
TCAs	1.59	(1.29 to 1.96)	1.20	(0.97 to 1.49)	1.19	(0.96 to 1.48)	1.11	(0.89 to 1.38)	1.09	(0.88 to 1.35)
SSRIs	1.02	(0.89 to 1.18)	0.92	(0.80 to 1.06)	0.91	(0.79 to 1.05)	0.85	(0.74 to 0.98)	0.84	(0.73 to 0.97)
Other antidepressants	1.55	(1.23 to 1.95)	1.32	(1.04 to 1.67)	1.31	(1.04 to 1.66)	1.24	(0.98 to 1.57)	1.21	(0.96 to 1.54)
Combined antidepressants	1.47	(0.75 to 2.89)	1.20	(0.61 to 2.36)	1.20	(0.61 to 2.35)	1.10	(0.57 to 2.15)	1.07	(0.54 to 2.09)
**Antidepressant drug**										
No current use	1.00		1.00		1.00		1.00		1.00	
TCAs:										
Amitriptyline	1.75	(1.32 to 2.33)	1.32	(0.99 to 1.75)	1.30	(0.98 to 1.73)	1.19	(0.89 to 1.59)	1.16	(0.87 to 1.54)
Dosulepin	1.31	(0.87 to 1.98)	0.97	(0.64 to 1.47)	0.96	(0.64 to 1.46)	0.94	(0.62 to 1.42)	0.93	(0.61 to 1.40)
Lofepramine	2.10	(1.28 to 3.46)	1.92	(1.17 to 3.15)	1.90	(1.15 to 3.12)	1.71	(1.03 to 2.82)	1.67	(1.01 to 2.76)
Trazodone	1.33	(0.55 to 3.21)	0.80	(0.30 to 2.16)	0.81	(0.30 to 2.18)	0.73	(0.27 to 1.96)	0.72	(0.27 to 1.94)
SSRIs:										
Citalopram	1.07	(0.90 to 1.28)	0.93	(0.77 to 1.12)	0.92	(0.76 to 1.10)	0.86	(0.72 to 1.04)	0.86	(0.71 to 1.03)
Escitalopram	1.33	(0.91 to 1.93)	1.11	(0.75 to 1.64)	1.10	(0.75 to 1.63)	1.07	(0.72 to 1.59)	1.06	(0.71 to 1.57)
Fluoxetine	0.86	(0.69 to 1.08)	0.82	(0.65 to 1.02)	0.81	(0.65 to 1.01)	0.75	(0.60 to 0.93)	0.74	(0.59 to 0.92)
Paroxetine	1.13	(0.77 to 1.66)	1.02	(0.69 to 1.50)	1.02	(0.69 to 1.50)	0.97	(0.66 to 1.43)	0.97	(0.66 to 1.43)
Sertraline	1.15	(0.80 to 1.65)	1.06	(0.73 to 1.53)	1.05	(0.72 to 1.51)	0.98	(0.68 to 1.42)	0.97	(0.67 to 1.40)
Others:										
Mirtazapine	1.73	(1.19 to 2.52)	1.34	(0.91 to 1.97)	1.34	(0.91 to 1.97)	1.25	(0.85 to 1.83)	1.20	(0.81 to 1.77)
Venlafaxine	1.45	(1.04 to 2.03)	1.34	(0.95 to 1.87)	1.33	(0.95 to 1.86)	1.28	(0.91 to 1.80)	1.27	(0.91 to 1.79)
All other antidepressants	1.02	(0.51 to 2.03)	0.82	(0.41 to 1.63)	0.81	(0.41 to 1.62)	0.75	(0.38 to 1.51)	0.73	(0.37 to 1.46)
Combined antidepressants	1.47	(0.75 to 2.89)	1.20	(0.61 to 2.36)	1.20	(0.61 to 2.35)	1.10	(0.56 to 2.14)	1.06	(0.54 to 2.08)

[1] Model A: adjusted only for age, sex, deprivation, ethnicity, year of diagnosis
[2] Model B: adjusted for age, sex, deprivation, ethnicity, year of diagnosis plus depression severity, smoking, alcohol
[3] Model C: adjusted for variables in model B plus coronary heart disease, diabetes, hypertension, stroke/TIA, rheumatoid arthritis, renal disease, statins, aspirin, antihypertensives, anticoagulants
[4] Full model: adjusted for confounders listed in footnote to Table 2

Table M Adjusted hazard ratios for myocardial infarction by antidepressant class, and individual drug over 5 years follow-up adjusted for different subsets of confounding variables.

	Unadjusted		Model A[1]		Model B[2]		Model C[3]		Full model[4]	
	Hazard ratio	95% CI	Adjusted hazard ratio	95% CI	Adjusted hazard ratio	95% CI	Adjusted hazard ratio	95% CI	Adjusted hazard ratio	95% CI
**Antidepressant class**										
No current use	1.00		1.00		1.00		1.00		1.00	
TCAs	1.83	(1.44 to 2.33)	1.33	(1.04 to 1.69)	1.27	(1.00 to 1.61)	1.23	(0.97 to 1.57)	1.20	(0.94 to 1.52)
SSRIs	1.02	(0.86 to 1.22)	0.91	(0.77 to 1.08)	0.90	(0.76 to 1.07)	0.85	(0.72 to 1.01)	0.85	(0.71 to 1.00)
Other antidepressants	1.39	(0.98 to 1.98)	1.07	(0.75 to 1.53)	1.01	(0.71 to 1.45)	1.00	(0.70 to 1.43)	1.00	(0.70 to 1.42)
Combined antidepressants	0.84	(0.27 to 2.59)	0.63	(0.20 to 1.92)	0.59	(0.19 to 1.82)	0.58	(0.19 to 1.78)	0.57	(0.18 to 1.75)
**Antidepressant drug**										
No current use	1.00		1.00		1.00		1.00		1.00	
TCAs:										
Amitriptyline	1.84	(1.28 to 2.62)	1.31	(0.92 to 1.86)	1.26	(0.88 to 1.79)	1.21	(0.85 to 1.72)	1.17	(0.82 to 1.66)
Dosulepin	1.79	(1.14 to 2.81)	1.27	(0.81 to 1.99)	1.21	(0.77 to 1.90)	1.20	(0.76 to 1.88)	1.17	(0.75 to 1.83)
Lofepramine	2.73	(1.54 to 4.84)	2.31	(1.30 to 4.11)	2.20	(1.24 to 3.91)	2.05	(1.16 to 3.64)	2.02	(1.14 to 3.59)
Trazodone	1.01	(0.25 to 4.06)	0.64	(0.16 to 2.56)	0.60	(0.15 to 2.40)	0.58	(0.14 to 2.33)	0.57	(0.14 to 2.30)
SSRIs:										
Citalopram	1.05	(0.83 to 1.33)	0.93	(0.73 to 1.18)	0.92	(0.73 to 1.18)	0.88	(0.69 to 1.12)	0.88	(0.69 to 1.12)
Escitalopram	0.89	(0.48 to 1.67)	0.83	(0.44 to 1.55)	0.83	(0.44 to 1.54)	0.79	(0.42 to 1.47)	0.77	(0.41 to 1.44)
Fluoxetine	0.89	(0.67 to 1.18)	0.80	(0.60 to 1.08)	0.79	(0.59 to 1.06)	0.73	(0.54 to 0.99)	0.73	(0.54 to 0.98)
Paroxetine	1.02	(0.60 to 1.73)	0.82	(0.47 to 1.41)	0.81	(0.47 to 1.41)	0.77	(0.44 to 1.33)	0.76	(0.44 to 1.31)
Sertraline	1.53	(1.01 to 2.31)	1.37	(0.90 to 2.09)	1.34	(0.88 to 2.04)	1.27	(0.84 to 1.94)	1.27	(0.84 to 1.94)
Others:										
Mirtazapine	2.21	(1.37 to 3.55)	1.43	(0.89 to 2.32)	1.31	(0.81 to 2.12)	1.32	(0.81 to 2.13)	1.31	(0.81 to 2.12)
Venlafaxine	1.06	(0.63 to 1.80)	0.93	(0.54 to 1.58)	0.90	(0.53 to 1.54)	0.89	(0.52 to 1.52)	0.89	(0.52 to 1.51)
All other antidepressants	0.72	(0.24 to 2.21)	0.55	(0.18 to 1.68)	0.54	(0.18 to 1.66)	0.53	(0.17 to 1.61)	0.52	(0.17 to 1.60)
Combined antidepressants	0.84	(0.27 to 2.59)	0.63	(0.20 to 1.93)	0.59	(0.19 to 1.82)	0.58	(0.19 to 1.78)	0.57	(0.18 to 1.75)

[1] Model A: adjusted only for age, sex, deprivation, ethnicity, year of diagnosis

[2] Model B: adjusted for age, sex, deprivation, ethnicity, year of diagnosis plus depression severity, smoking, alcohol

[3] Model C: adjusted for variables in model B plus coronary heart disease, diabetes, hypertension, stroke/TIA, rheumatoid arthritis, renal disease, statins, aspirin, antihypertensives, anticoagulants

[4] Full model: adjusted for confounders listed in footnote to Table 5

Table N Adjusted hazard ratios for stroke/TIA by antidepressant class, and individual drug over 5 years follow-up adjusted for different subsets of confounding variables.

	Unadjusted		Model A[1]		Model B[2]		Model C[3]		Full model[4]	
	Hazard ratio	95% CI	Adjusted hazard ratio	95% CI	Adjusted hazard ratio	95% CI	Adjusted hazard ratio	95% CI	Adjusted hazard ratio	95% CI
**Antidepressant class**										
No current use	1.00		1.00		1.00		1.00		1.00	
TCAs	1.98	(1.56 to 2.52)	1.40	(1.10 to 1.77)	1.35	(1.06 to 1.72)	1.31	(1.03 to 1.67)	1.24	(0.98 to 1.58)
SSRIs	1.30	(1.12 to 1.51)	1.18	(1.01 to 1.37)	1.16	(1.00 to 1.36)	1.11	(0.96 to 1.29)	1.09	(0.93 to 1.27)
Other antidepressants	1.71	(1.30 to 2.25)	1.33	(1.01 to 1.77)	1.29	(0.97 to 1.71)	1.27	(0.96 to 1.68)	1.20	(0.91 to 1.60)
Combined antidepressants	2.59	(1.47 to 4.55)	1.84	(1.02 to 3.31)	1.77	(0.98 to 3.21)	1.71	(0.95 to 3.08)	1.54	(0.86 to 2.78)
**Antidepressant drug**										
No current use	1.00		1.00		1.00		1.00		1.00	
TCAs:										
Amitriptyline	2.27	(1.69 to 3.06)	1.56	(1.16 to 2.11)	1.51	(1.12 to 2.04)	1.44	(1.07 to 1.94)	1.35	(1.00 to 1.82)
Dosulepin	1.81	(1.20 to 2.72)	1.23	(0.81 to 1.85)	1.19	(0.79 to 1.80)	1.20	(0.79 to 1.80)	1.16	(0.77 to 1.76)
Lofepramine	2.27	(1.24 to 4.15)	1.94	(1.07 to 3.53)	1.90	(1.04 to 3.44)	1.85	(1.01 to 3.36)	1.75	(0.96 to 3.19)
Trazodone	0.77	(0.19 to 3.06)	0.52	(0.13 to 2.00)	0.49	(0.13 to 1.89)	0.46	(0.12 to 1.76)	0.43	(0.11 to 1.66)
SSRIs:										
Citalopram	1.24	(1.02 to 1.52)	1.14	(0.93 to 1.39)	1.12	(0.92 to 1.37)	1.08	(0.89 to 1.32)	1.06	(0.87 to 1.30)
Escitalopram	1.15	(0.70 to 1.88)	1.04	(0.64 to 1.71)	1.03	(0.63 to 1.68)	1.00	(0.61 to 1.64)	0.97	(0.59 to 1.59)
Fluoxetine	1.36	(1.11 to 1.65)	1.26	(1.03 to 1.54)	1.24	(1.01 to 1.52)	1.16	(0.95 to 1.42)	1.13	(0.93 to 1.39)
Paroxetine	1.21	(0.78 to 1.88)	1.00	(0.64 to 1.57)	0.99	(0.63 to 1.56)	0.97	(0.62 to 1.53)	0.95	(0.61 to 1.50)
Sertraline	1.55	(1.06 to 2.25)	1.37	(0.94 to 1.99)	1.35	(0.93 to 1.96)	1.29	(0.89 to 1.88)	1.26	(0.86 to 1.83)
Others:										
Mirtazapine	2.23	(1.51 to 3.28)	1.56	(1.05 to 2.33)	1.48	(0.99 to 2.20)	1.44	(0.96 to 2.15)	1.35	(0.90 to 2.02)
Venlafaxine	1.33	(0.88 to 2.03)	1.11	(0.72 to 1.73)	1.09	(0.70 to 1.69)	1.09	(0.71 to 1.70)	1.05	(0.67 to 1.63)
All other antidepressants	1.47	(0.73 to 2.96)	1.13	(0.56 to 2.26)	1.11	(0.55 to 2.21)	1.09	(0.54 to 2.19)	1.00	(0.50 to 2.02)
Combined antidepressants	2.59	(1.47 to 4.55)	1.84	(1.02 to 3.31)	1.78	(0.98 to 3.21)	1.71	(0.95 to 3.09)	1.55	(0.86 to 2.78)

[1] Model A: adjusted only for age, sex, deprivation, ethnicity, year of diagnosis
[2] Model B: adjusted for age, sex, deprivation, ethnicity, year of diagnosis plus depression severity, smoking, alcohol
[3] Model C: adjusted for variables in model B plus coronary heart disease, diabetes, hypertension, rheumatoid arthritis, renal disease, statins, aspirin, antihypertensives, anticoagulants
[4] Full model: adjusted for confounders listed in footnote to Table 6
